# The global invasion risk of rice yellow stem borer *Scirpophaga incertulas* Walker (Lepidoptera:Crambidae) under current and future climate scenarios

**DOI:** 10.1371/journal.pone.0310234

**Published:** 2025-03-05

**Authors:** Shravani Sanyal, A.V.M. Subba Rao, H. Timmanna, G. Baradevanal, Santanu Kumar Bal, M.A. Sarath Chandran, P.R. Shashank, V.K. Singh, P.K. Ghosh

**Affiliations:** 1 ICAR-National Institute of Biotic Stress Management, Raipur, Chhattisgarh, India; 2 ICAR- Central Research Institute for Dryland Agriculture, Hyderabad, Telangana, India; 3 ICAR-National Bureau of Agricultural Insect Resources, Bengaluru, Karnataka, India; 4 ICAR-Indian Agricultural Research Institute, New Delhi, Delhi, India; Central Rice Research Institute: ICAR - National Rice Research Institute, INDIA

## Abstract

Rice yellow stem borer, *Scirpophaga incertulas* (Walker) (Lepidoptera: Crambidae) is a serious pest, that causes significant yield loss (10-40%) to rice crop in India and other parts of the world. This study emphasizes the prediction of the potential invasion risk, distribution, and further spread of *S. incertulas* during current and future climate change scenarios in India and the world. The pest identity was confirmed with morphological taxonomy, and the possible habitat distribution and further spread in future climate scenarios were modelled using the MaxEnt algorithm. The climate niche for *S. incertulas* was also established by analyzing the correlation between the pest occurrence data of 143 locations in India and seven bioclimatic variables *viz*., bio01, bio02, bio03, bio05, bio12, bio13, and bio15, were chosen for predicting the distribution of *S. incertulas*. The model performance was good as it exhibited a strong Receiver Operating Characteristic Curve value of 0.949. Based on the Jackknife test, the Bio 13 (precipitation of the wettest month), exhibited the highest gain value and emerged as the primary abiotic factor exerting influence on the potential habitat distribution of this borer. The study demonstrated that bioclimatic variables annual mean temperature (30 °C), and annual precipitation (10-700 mm) favour its multiplication, infestation, and further spread to new areas. As the anticipated habitat range of *S. incertulas* is of considerable importance for researchers and other stakeholders involved in plant protection, the data generated here may be useful for researchers, policymakers, and farmers for designing better management strategies to mitigate this pest and curtail its spread to new rice growing areas in a rapidly changing global environment.

India ranks second in production as well as consumption of rice on a global scale. Total production during 2022-23 is estimated at a record 1357.55 lakh tonnes [1]. Due to the changing climate, there are various abiotic and biotic stresses causing significant yield loss in rice crops. Among the biotic stresses, the yellow stem borer, *Scirpophaga incertulas* is a key pest of rice that is widely distributed in most of the rice-growing agroclimatic zones of India [2,3] and in other Asian countries *viz.,* Afghanistan, Bangladesh, Borneo, China, Japan, Java, Myanmar, Nepal, Philippines, Singapore, Sri Lanka, Sulawesi, Sumatra, Thailand, Vietnam, West Malaysia [4].

The larvae bore and feed the internal content which results in the drying of growing tillers, this condition is called a “dead heart.” During the reproductive stage, when plants are subjected to attack, larvae consume the meristem, resulting in the emergence of empty or chaffy, whitish panicles known as ‘white ear heads’. The *S. incertulas* cause yield losses to the tune of 27 to 34% every year in non-basmati rice [5] and 10-13% yield loss reported in basmati rice [6]. Due to its internal feeding habit, its damage diagnosis is not possible till the panicle appearance. Researchers fromthe National Rice Research Institute Cuttack estimated that for every 1% increase in white earheads, and yields were reduced by 2.2% [7,8].

The occurrence, distribution, and damage of *S. incertulas*are primarily driven by abiotic factors, which cause a substantial impact on its overall growth and development [9]. Congenial microclimate in rice fields enhances the *S. incertulas* incidence [10]. Climate change with variability in weather conditions has a direct impact on the behaviour of *S. incertulas* and its natural enemies in the rice ecosystem [11]. Three or more generations occur in a season, the adult stage is characterized by the presence of the moth’s activity, and can fly short distances, they can travel 8-16 km, if carried by wind [12].

A solitary female lay 100 to 200 eggs, which are enveloped by pale brown hairs of anal tufts. The varieties with a greater number of tillers [4,13–17], favour the infestation, the recently emerged larvae have the tendency to engage in external feeding for a certain duration. Subsequently, they proceed to penetrate the stems, by targeting the upper tender nodes, and gradually towards the plant's base [18]. The tillering to flowering stages are susceptible stages for *S. incertulas*infestation. During off-season *S. incertulas* will survive on weed hosts like Barnyard grass (*Echinocloa*sp.) and common millet (*Panicum miliecium*) [19]. Asynchrony of planting date is another major factor that increases damage, especially in late-planted crops [20].Yellow stem borerisdistributed in tropical South and Southeast Asia. Its incidence is most predominant in tropical lowland rice and deep-water rice. *S. incertulas* is one of the major or most serious rice pests in Bangladesh, India, Malaysia, Myanmar, Pakistan, Philippines, Sri Lanka, Thailand, Vietnam and IndonesiaIt is also a predominant species in Taiwan, south Japan, south China and south Nepal (PlantwisePlus Knowledge Bank, 2022).

There are various distribution models are used to estimate the occurrence of a species based on habitat attributes such as climate, soils, and land usepatterns. These models are then spatially projected to represent the modelled species’ potential habitat and provide an objective tool to understand the species distribution. Over the past few decades, statistical and technical capabilities have progressed to a level where we now have a variety of techniques for predicting the potential distribution of a given species [21,22,23]. These techniques use field observations of a species (*i.e.*, presence-absence data or presence-only data) as a dependent variable, while independent or predictive variables represent environmental conditions (e.g., elevation, minimum temperature, mean monthly precipitation). MaxEnt is a general-purpose machine learning method with a better performance in predicting species distribution [14,24]. Maxent has been applied to a wide range of studies, including those related to discovering rare species [17], predicting the spread of invasive species *viz*., Invasive chilli thrips, *T. parvispinus,* rugose whitefly, *A. rugioperculatus* [25,26], prediction of pest free areas for the quarantine pest like mango weevil, *Sternochetus mangiferae*, Red-banded mango caterpillar, *Deanolis sublimbalis* [2,27] and assessing plant disease riskslike mango wilt, *Ceratocystis fimbriata* [28].

The various abiotic factors strongly influence the occurrence and distribution of stem borer population. However, the development of stem borer life stages is strongly driven by temperature. Cooler temperatures and day length changes induce diapause in mature larvae. The habitats and survival strategies of insects are strongly dependent on temperature because they are cold–blooded. Therefore, temperature is probably the most important environmental factor influencing their behavior, distribution, development, survival and reproduction [29]. An analysis conducted by IPCC and Pachauri et al [30,31], indicated that the Earth’s climate has warmed by approximately 0.74°C and it is also projected to increase by 1.1 to 6.4°C at the end of the 21^st^ century. Such an increase in temperature will affect the physiological process and cause faster development and possibly result in more generations in a season [32] All the developmental stagesof rice brown plant hoppers were affected by their thermal environment between 31-38°C as compared to 26°C [33], and eggs could respond early, as soon as the embryonic development starts. The potential rate of development in insect population is strongly dependent on temperature and their survival is impaired at temperature extremes [30]. The projected warming will certainly increase the exposure and survival opportunities of insects and other ectothermic species to high temperatures, exceeding their upper physiological limits. It is important to understand the response of insects to changes in temperature, as the temperature is important for their survival and distribution. Hence, in this study we hypothesize that the risk of *S. incertulas* will significantly increase under future climate scenarios compared to the current climate, driven by changes in temperature and precipitation patterns that expand suitable habitats and enhance the pest’s survival and reproductive capacities.

## 2. Materials and methods

### Study locations and data

An extensive roving survey was conducted to assess the occurrence, infestation level of *S. incertulas*and its distribution in major rice-growing districts of different states *viz*., West Bengal, Chhattisgarh, Haryana, Uttar Pradesh, Assam, Telangana, Maharashtra, Kerala, and Karnataka during 2018-2023.The observation was recorded from 20 plants from each field (two fields in each district from each state were selected and data of pest infestation at the peak infestation was recorded, similarly adult mothactivity was monitored by installing light traps (1trap/5 acre), and the number of moths trapped was recorded. The GPS coordinates of these locations were collected. In addition to this, the global occurrence records were acquired utilizing data mining techniques applied to available literature and authenticated databases such as the Centre for Agriculture and Bioscience International [34] and the Global Biodiversity Information Framework [35].

### Morphological investigation of *S. incertulas*

The samples of adult moths, *S. incertulas* were collected and preserved in brown paper covers for taxonomic examination. The morphological taxonomy was carried out at the National Pusa Collection, ICAR-Indian Agricultural Research Institute in New Delhi.

More than 50 specimens of males and females were used for slide mount preparation, the genitalia is the main taxonomic character that was used for differentiating species of *Scirphophaga* genus. The slide mounts with good specimen position and better claritywere used for taxonomic description.

Key morphological characters viz., length of labial palpi with respect to the diameter of the eye, venation of wings, colour and markings on wings, male genitalia characters viz., the shape of gnathos, uncus, valva, presence of costal or subteguminal processes, the shape of the aedeagus. And female genitalia characters like signum, and shape of the bursa copulatrix were used. The genitalia slide of moths were prepared by separating the abdomen from a small hitch at its base. The slide mount preparation and key out of morphological characters was done by the protocol and keys structured by Dey and Shashank [36]. The adult moths were photographed using a Canon 80D with a 110 mm macro lens. The slides of male and female genitalia were photographed with a digital camera Leica DFC 425C on a Leica 205FA stereo microscope with auto-montage. The taxonomic characters were identified with the help of genitalia structures using taxonomic keys [36]. The slide mounts (10 no.) were deposited in the National Pusa Collection, Indian Agricultural Research Institute, New Delhi, India (NPC, Repository register numbers (RRS No.) 2022-38, 2022-39, 2022-40, 2022-41, 2022-42, 2022-43, 2022-44, 2022-45, 2022-46, 2022-47).

### 2.2 Assessment of invasion risk of *S. incertulas*

**Fig 1:**
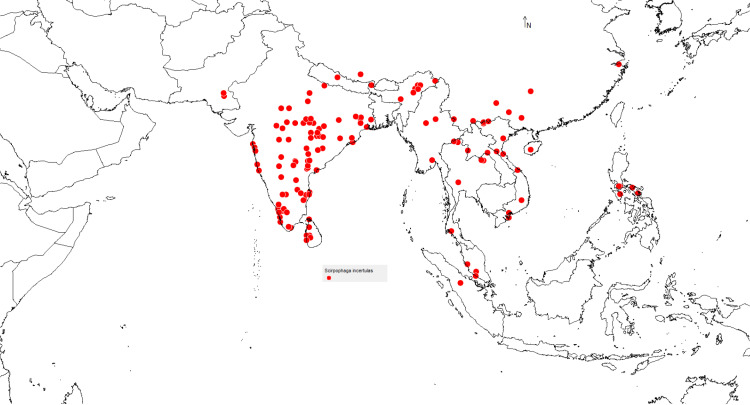
Occurrence records of *S. incertulas* in the world.

The investigation utilized a total of 143valid existence points (Supporting information S1 Table). In order to overcome uncertainty and sample bias, the ‘spThin’ R package was employed to eliminate duplicate records and surrounding occurrence locations [37,27].These places of *S. incertulas* dominance were used as occurrence points to identify the present and future infestation and invasion risk mapped on India and world maps using open-access software DIVA-GIS version 7.5 [38] and shape files for visualisation were downloaded from Natural Earth website (https://www.naturalearthdata.com/) (Fig 1).

#### 2.2.1 Bioclimatic variables data.

A total of 19 bioclimatic variables data necessary for conducting various analyses such as ecological niche characterization, potential geographic distribution prediction, and assessment of climate change effects on the future spread and distribution of *S. incertulas*, were downloaded from the worldclim database (http://www.worldclim.org). To assess the possible distribution under present climatic circumstances, we utilized a reference dataset consisting of long-term time series spanning from 1950 to 2000. This dataset was obtained from a global network and was characterized by a spatial resolution of 2.5 arc-minutes, which corresponds to around 4.5 km at the equator. It is not always the case that all 19 bioclimatic variables are solely responsible for identifying the possible habitat distribution of the insect species being investigated. Therefore, Pearson's correlation coefficient was utilized to categorize and eliminate factors with high correlation in each pair wise comparison of the 19 climatic variables. According to Wei et al. [39], in cases where two variables exhibited a Pearson's coefficient value of r ≥ 0.85, only one variable was chosen for model development based on its relative importance in determining *S. incertulas* distribution and its predictive power. The selection was made considering factors such as percent contribution and jackknife training gain. The probable habitat distribution of *S. incertulas* was analysed by further processing a total of seven environmental factors, namely Annual mean temperature (bio01), Mean Diurnal Range of temperature (Mean of monthly (Tmax - Tmin)) (bio02), Isothermality (bio03 =  bio2/bio7), Max Temperature of Warmest Month (bio05), Mean Diurnal Range of temperature Mean of monthly (Tmax - Tmin) (bio02), Precipitation of wettest month (bio13) and Precipitation Seasonality (Coefficient of Variation) (bio15)for predicting the distribution of *S. incertulas* (Table 1).

**Table 1:** Bioclimatic variables used for the study.

Bioclimatic variable	Percent contribution	Permutation importance
Annual mean temperature (bio 01)	29.2	68.5
Mean Diurnal Range (Mean of monthly (max temp - min temp)) (bio 02)	7.2	6.5
Isothermality (bio03 = bio2/bio7)	10.9	15.2
Max Temperature of Warmest Month (bio05)	6.5	0.3
Annual Precipitation (bio12)	1.4	0.1
Precipitation of wettest month (bio13)	39.2	9.3
Precipitation Seasonality (Coefficient of Variation) (bio15)	5.5	0.0

#### 2.2.2 Future climate data.

Downscaled data of future climate for 2030, 2050, 2070, and 2080 has been obtained from the Climate Change Agriculture and Food Security (CCAFS) portal (https://www.ccafs-climate.org/). The General Circulation Model, Geophysical Fluid Dynamics Laboratory (GFDL) of the Intergovernmental Panel on Climate Change Assessment Report 5 (AR5) was used to simulate the conditions of Representative Concentration Pathway 2.6 (RCP 2.6) in the years 2030, 2050, 2070, and 2080. RCP 2.6 shows a low emission scenario with lower greenhouse gas emissions, resulting in a peak radiative forcing of 3.1 W/m² before declining to 2.6 W/m² by 2100.RCPs 4.5, 6, and 8.5 were also predicted for 2030, 2050, 2070, and 2080, correspondingly.

### 2.3 Model settings

The software package MaxEnt version 3.4.4 was employed to conduct species distribution modelling. The MaxEnt algorithm was selected as it utilizes species presence data only, in conjunction with background information. The model was configured with specific settings, including a convergence threshold of 0.01, a maximum iteration limit of 500, and a maximum number of background points set at 10,000, to execute the model. Minimum training presence was selected in apply the threshold rule. The jackknife test was conducted to determine the relative importance of each variable in the creation of the model, based on background data. The validation of the model's performance was conducted by assessing the area under the curve (AUC) of the receiver operating characteristic (ROC) using a random selection of 25% of the locales for testing purposes. AUC values that are less than 0.5 are commonly considered indicative of random prediction. According to Pearson et al. ([40]), values of 0.5 and 0.7 are indicative of substandard model performance, whereas values ranging from 0.7 to 0.9 suggest an acceptable degree of performance. Additionally, values exceeding 0.9 are indicative of a high level of model performance. The distribution maps were processed according to the methodology outlined by previous authors ([27,26] ). Suitability maps were prepared using open-access software DIVA-GIS version 7.5. The shape files required for the visualisation of maps were downloaded from the Natural Earth website (https://www.naturalearthdata.com/) (ne_10m_admin_0_map_units.shp). And the data is available under a CC BY 4.0 license

## 3. Results

The pest data from regular surveysand monitoring of *S. incertulas* of five consecutive crop-growing *kharif* seasons from 2018 to 2023and the visual observation and light trap catch data revealed that *S. incertulas* activity was observed from vegetative to reproductive stages of rice crops, and it was prevalent in all nine major rice growing states selected for the study. Irrespective of rice crop varieties, comparatively higher-level infestation was observed in low-land crops (irrigated) during the *kharif* season. Among the nine states, a higher level of infestation (% white earhead)and more moths were trapped in light trapsat Haryana (16%; 23.8), followed by West Bengal (13%;26.32), Uttar Pradesh (12.08%;19.44), Assam (11.70%;15.3), Chhattisgarh (9.67%;13.4), Maharashtra (8.77%;12.7), Telangana (10%;8.9), Kerala (9.50%;7.8) and Karnataka (9.23%;7.4) (Table 2).

**Table 2:** Occurrence of *S. incertulas* in major rice-growing states of India (2018-2023).

State	Districts	Infested White ear head (%)	Light trap catches (Moths/week)
Haryana	Gurugram (Patuadi, Dadawas, Mouzabad)	16.00	23.8
West Bengal	Mohanpur, Purba Medinipur, Bankura, Jhargram, Purulia and Hooghly	13.00	26.32
Uttar Pradesh	Hapur (Peernagar Soodna) Lucknow	12.08	19.44
Chhattisgarh	Raipur, Balod, Balrampur, Bastar, Bemetara, Bilaspur, Dantewada, Dhamtari, Durg, Gariyaband and Jashpur	9.67	13.36
Assam	Majuli, Dhemaji, Jorhat (Dibrugarh, Golaghat and Sibsagar	11.70	15.28
Maharashtra	Dapoli, Palghar, Thane, Raigad, Ratnagiri and Sindhudurg	8.77	12.72
Telangana	Rangareddy, Narayanpet	10.00	8.92
Kerala	Thrissur, Malappuram, Kuttanad, Ernakulam, Wayanad and Palakkad	9.50	7.8
Karnataka	Mandya, Koppal, Bellary, Raichur	9.23	7.4

### 3.1 Taxonomic description of *S. incertulas*

**Fig 2:**
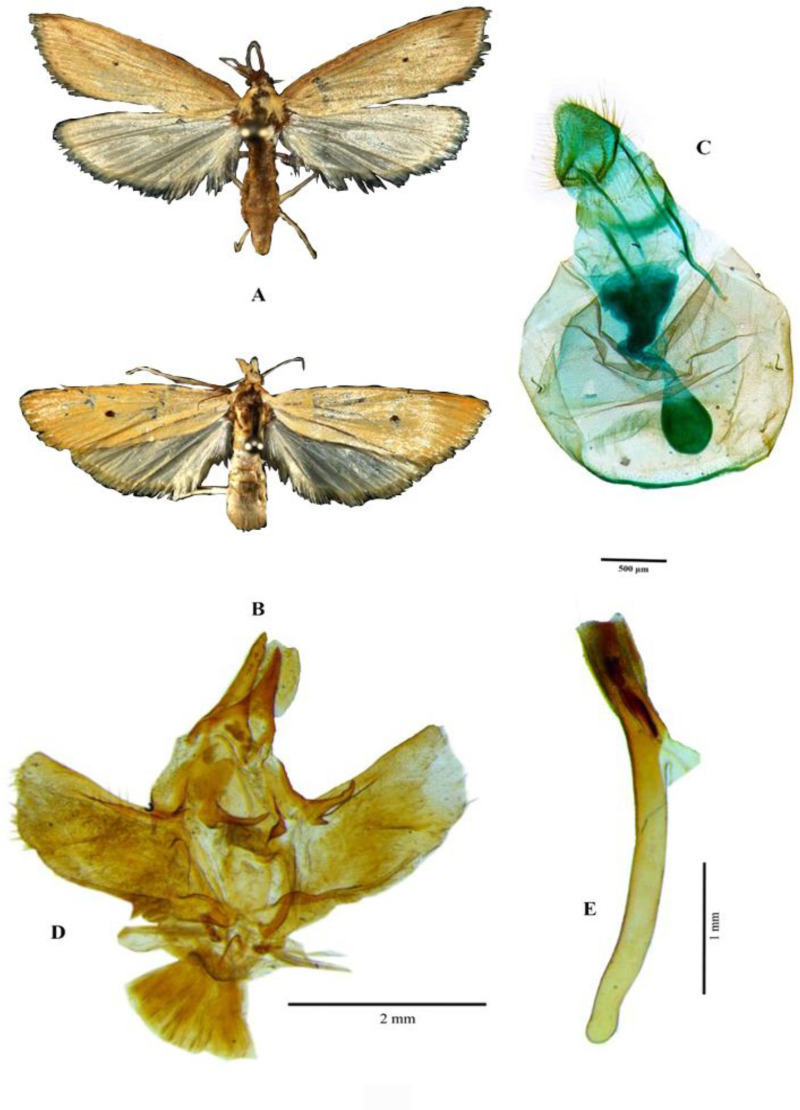
Yellow stem borer, *Scirpophaga incertulas* (Walker, 1863) Male (A) Female (B) Female genitalia (C) Male genitelia (D), Aedeagus (E).

Adult males of *S. incertulas* are light brown. Forewings have a tinge of brown color while hind wings are white. Male adults are relatively bigger as compared to female adults. The forewings of the female borer are yellowish and the hind wings are pale white in colour (Fig 2). *S. incertulas* resembles *S. innotata*, however, *S. innotata* has white wings without any spots while in *S. incertulas*, and the wings are ocherous to pale yellow and have a dark fuscous spot. Additionally, in male genitalia of *S. innotata*, the sub-teguminal process is with a single spine whereas in *S. incertulas* it is a double spine.

### 3.2 Dominant role of bioclimatic variables for the spread of *S. incertulas*

**Fig 3:**
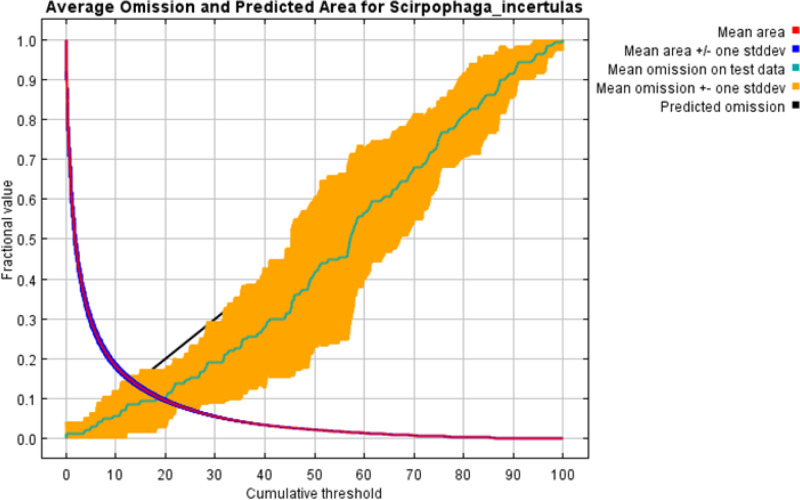
Test omission rate and predicted area.

**Fig 4:**
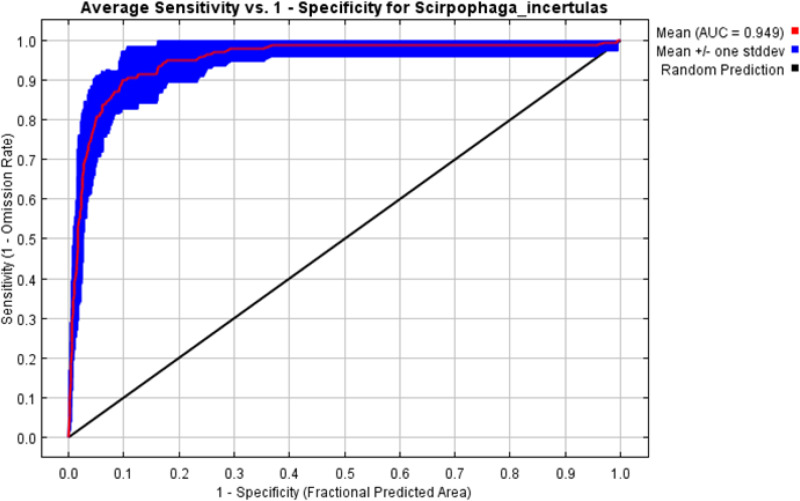
Receiver operating characteristic (ROC) curve for the *S. incertulas.*

**Fig 5:**
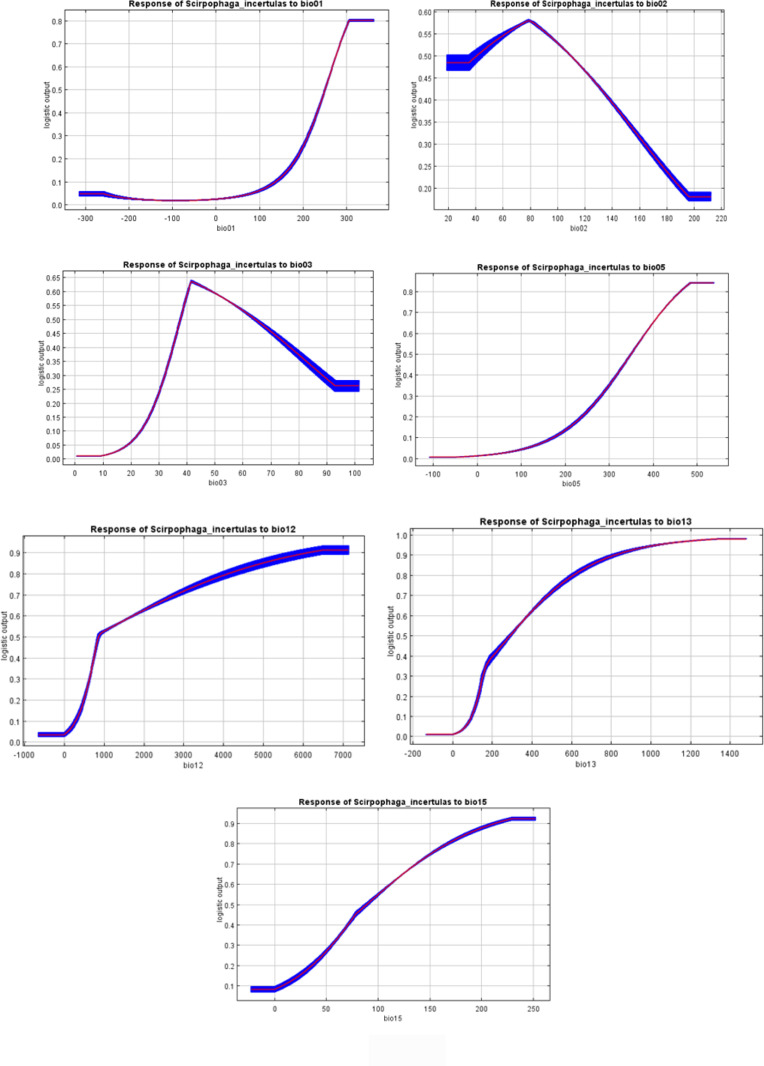
Response curves for the *S. incertulas* (predicted probability of presence changes as each environmental variable is varied).

**Fig 6:**
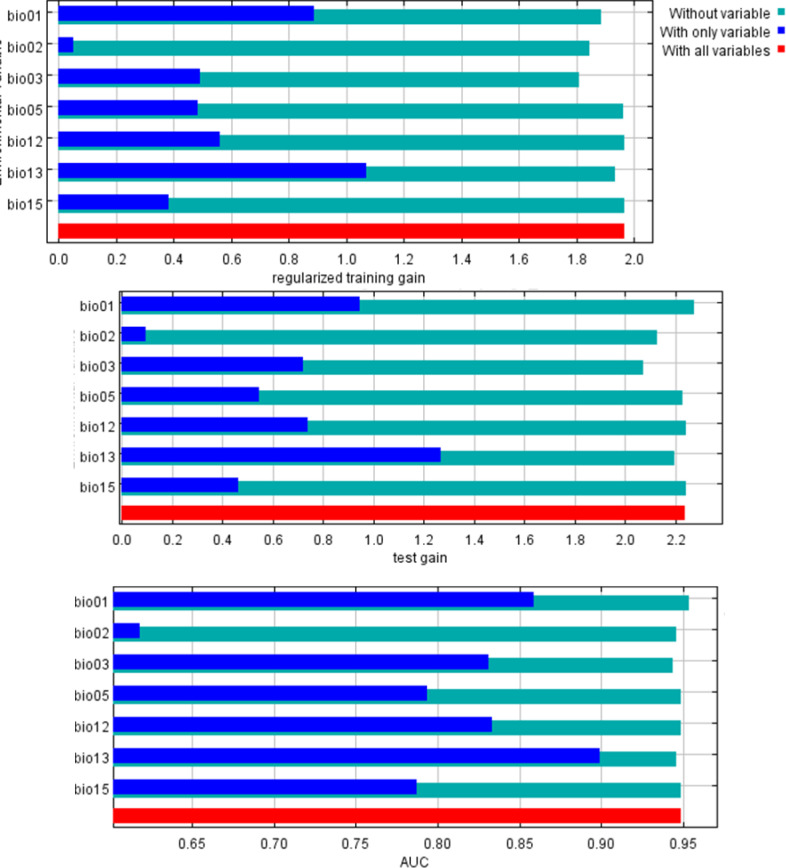
Jackknife test of variable importance a) regularized training gain b) test gain c) AUC.

In the MaxEnt model, test omission, Receiver Operating Characteristic (ROC) curve, and Area Under the ROC Curve (AUC) are crucial metrics for evaluating the model's performance and predictive accuracy. The spread of *S. incertulas* matched close to the test omission rate. The test omission rate was near to the predicted omission (Fig 3) and the predicted omission was aligned with the receiver operating characteristic (ROC) curve. Low test omission rate indicates good model performance, as most test presence points are correctly predicted by the model. The value in an area under the curve (AUC) was 0.949 (Fig 4) suggesting good predictive performance, indicating that the model effectively distinguishes between species presence and absence across a range of threshold values for habitat suitability of *S. incertulas.* It was clearly shown that bioclimatic variables temperature and precipitation played a pivotal role in the niche suitability of *S. incertulas*. The relationship between key bioclimatic variables suggests the likelihood of more occurrences of *S. incertulas*. The bioclimatic variables *viz*., annual mean temperature (bio01 = 30°C), mean diurnal temperature range (bio02 = 8°C), Isothermality (bio03 = 4°C), wettest month precipitation range (bio13 = 10-140 mm), the maximum temperature of the warmest month (bio05 = 50°C), precipitation seasonality (bio15 = 25mm), and mean annual precipitation (bio12 = 10-700 mm) (Fig 5).The environmental variableprecipitation of the wettest month (bio13), yielded the greatest gain indicating its significant role in providing valuable information. The variable mean diurnal temperature range(bio02), exhibited the most significant impact on the drop-in gain resulting from exclusion, indicating that contained the highest amount of unique information as compared to the other variables (Fig 6).

Maybe there are prediction uncertainties with correlative niche models (1429). These uncertainties are predominantly due to the consistency of data on pest prevalence, sampling bias, spatial data layer resolution, species characteristics, and spatial autocorrelation. Nevertheless, several calibrations can be done, which can have a significant impact on the model and thus on its accuracy [41,42]. These calibrations include the selection of background points and extent, the value of the regularization multiplier, and the selection of feature types [43]. In the modelling process, we took utmost care in model calibration because of these potential pitfalls and thus developed predictive models that were consistent with the current known distribution of the species in terms of satisfactory validation of the predicted distribution of the pest.

### 3.3 Climate change impact on the spread of *S. incertulas* under current scenario

The study revealed that the spatial distribution of *S. incertulas*was influenced by various climatic factors. The precipitation of the wettest month (39.2%), annual mean temperature (29.2%), isothermality (10.9%), mean diurnal range (7.2%), maximum temperature of the warmest month (6.5%), precipitation seasonality (5.5%), and annual precipitation (1.4%) contributed significantly to determining its distribution (Table 1). In the analysis of the bioclimatic variables’ permutation importance, the annual mean temperature (bio 01) exhibited the highest level of significance in predicting the distribution of *S. incertulas* with an accountability of 68.5% of the overall contribution in the model.

**Fig 7:**
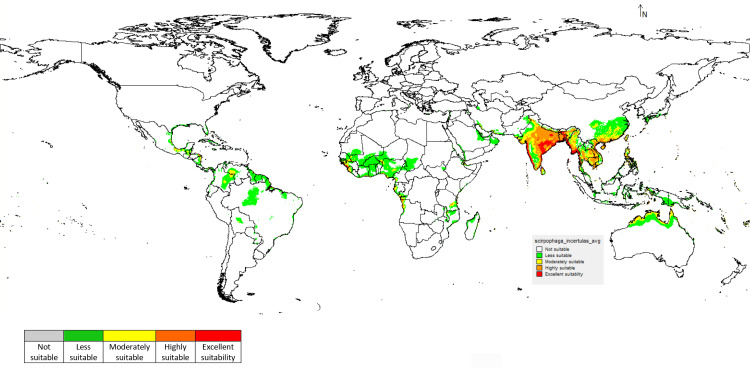
Prediction of *S. incertulas* under current scenario.

The study suggested that based on the habitat suitability *S. incertulas* occurrence was likely to be more in favourable areas of India and Southern parts of Bangladesh. In India, some parts of Odisha (Angul, Boudh, Cuttack, Jajpur), Chhattisgarh (Raipur, Bilaspur, Raigarh, Baloda Bazar), West Bengal, Assam and the western coast of India provided a congenial environment for its distribution. In other locations, appropriateness ranges from low to moderate suitability (Fig 7). Other states like Gujarat, Rajasthan, Punjab, Haryana, Delhi, Tamil Nadu and Andhra Pradesh are indicated as moderately suitable locations. Worldwide this pest is moderately suited to Thailand, Laos, Cambodia, Vietnam, Malaysia and western parts of China. Whereas Brazil, Nigeria, Niger, Chad, Sudan and Mali are considered less suitable for the occurrence and distribution of *S. incertulas*.

### 3.4 Spread of *S. incertulas* under future climatic scenario

**Fig 8:**
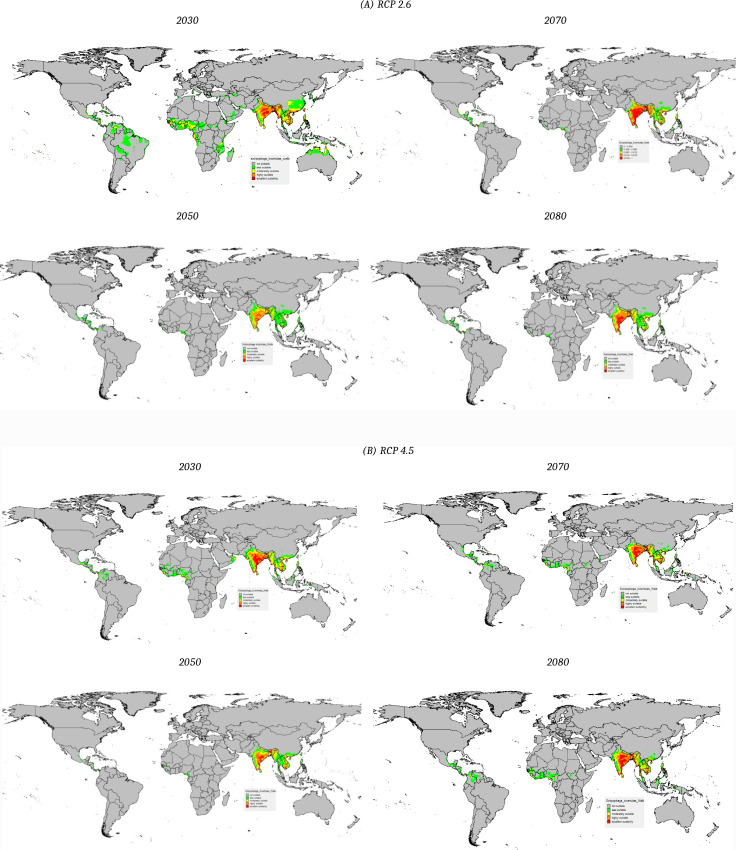
Prediction of S. incertulas distribution under future climate scenarios (A (RCP 2.6), B (RCP 4.5), C (RCP 8.5)) using GFDL model.

Future predictions by General Circulation Model (GCM) of Geophysical Fluid Dynamics Laboratory (GFDL) depicted that under the Representative Concentration Pathway (RCP) 2.6 scenario, *S. incertulas* infestation will not be the major threat in the upcoming year 2050 (Fig 8). However, in 2030, 2070 and 2080more infestation were predicted in the regions of Jharkhand, West Bengal, Chhattisgarh and Odisha. A similar trend was also observed for RCP 4.5 and 8.0.

## 4 Discussion

The changing climate leads to congenial local weather conditions like favourable temperature, moderate precipitation, and sunshine hours which are considered crucial factors for *S. incertulas* infestation [44]. The mapping of the occurrencepoints has revealed the higher infestation in major rice-growing states viz., Odisha, Chattishgarh, West Bengal, Assam,and the Western coast of India.According to the MaxEnt model, in the current scenario, 30°Cmean temperature is congenial for infestation, this finding was supported by Devi and Verma [45], who recorded more maximum moths in light traps and pheromone traps when the temperature ranged between 28 to 32°C in rice fields at Telangana. The *S. incertulas*multiplication was predicted more when the monthly precipitation ranges between 10 to 140mm and the maximum temperature of the hottest month are 50°C. These results are in conformity with the previous findings of Singh et al. [46], who reported the maximum incidence of *S. incertulas* during the optimum temperature (24-29°C), Relative humidity (90-95%), and Sunshine hrs (14-16 hrs). Similarly, Jasrotia et al. [47] recorded a maximum infestation of *S. Incertulas* at 27-32°C during Kharif. The model predicted the mean diurnal temperature range of 8°C to be crucial in the proliferation of *S. incertulas.*Round the year, a warm and moist temperature creates humid atmospheric air which in turn favors microclimate in rice fields for *S. incertulas* multiplication and infestation. the peak incidence was noticed during August (vegetative stage),and October (reproductive stage) [48]. Similarly in another study,the wettest month had a higher level of *S. incertulas* infestation when there was comparatively more precipitation during the Second week of August [12].

Low values of omission rates were indicative of precise mapping of the habitat niche,especially in the case of invasive species [49,50,51]. The model performed well andwas depicted by the Area Under Curve (AUC) of the Receiver Operating Characteristic (ROC) depicting a value of 0.949 which comes under excellent fit. In a similar line high-value AUC in ROC predicts the areas of infestation and spread accurately for various invasive pests in multiple locations [52,16,26]. ROC Curve represents the most suitable parameter in terms of model performance [15,4,53]. The regularizationmultiplier was kept as 10 in the model to avoid any underestimation or overestimation. The present study was an approach to delineate the hotspots and suitable areas of *S. incertulas* in India and the world depicting the environmental conditions that favour its spread. But in the present context up to 2030, this pest will cause substantial damage, and later it will decline. These results were supported by the findings [54,3,10], who documented that the future *S. incertulas*will not be a major challenge because the pupa stage of will not survive under extreme temperatures in all climatic scenarios. Similarly, the high mortality rates of its closer group stem borer, *Chilo partellus* observedat a temperature range between 18°C to 35°C. According to Stevens climatic variability hypothesis [55] the thermal tolerance of an insect is directly proportional to climate variability. The temperature between 20 and 32°C is considered as most suitable and a good intrinsic rate (0.108 and 51.091) was observed at 30°C [46]. Hence the present study has dealt with the regions that are presently under infestation of *S. incertulas* and also the areas that can become suitable habitats if favourable conditions for the pest exist.

The results of this study would be useful for different stakeholders of rice farming *viz*., rice growers, plant protectionists, and policymakers for designing sustainable management strategies as well as appropriate quarantine practices to mitigate this pest and contain its further spread. Here we employ sophisticated climate modelling methodologies to forecast the probable alterations in the habitat range of the *S. incertulas*, considering several climate change scenarios.

## 5. Conclusions

This study assessed the suitable and unsuitable areas of *S. incertulas* Walker in India and other Asian countries by MaxEnt model. Weather parameters viz., the temperature around 30°C and precipitation between 10 mm to 700 mm considered favorable factors and are directly connected to its distribution and further spread. The graphs and spatiotemporal variability maps generated in this study would certainly provide an insightful understanding of the climate factorsthat affect this pest infestation, distribution, and further spread to new areas. The data generated here would also have apractical utility in effectively monitoring and facilitating its risk assessment programs, and initiating real-time interventions to mitigate this yellow stem borer, *S. incertuals* further spread to other rice-growing areas,where it is absent.

## Supporting information

S1 Table Table showing occurrence points of Scirpophaga incertulas Walker. (DOCX)

S1 File SupplimentaryCompressed (zipped) Folder. (ZIP)
