# Supplementary material for: The global invasion risk of rice yellow stem borer Scirpophaga incertulas Walker (Lepidoptera:Crambidae) under current and future climate scenarios
Source: PLoS One. 2025 Mar 5;20(3):e0310234. doi: 10.1371/journal.pone.0310234 (PMC11882091; doi:10.1371/journal.pone.0310234)
Supplement: S1 Table — (DOCX) [file pone.0310234.s001.docx]

**Supplementary Table 1: Occurrence points of *Scirpophaga incertulas* Walker**

| S.N. | State/ District | country | Latitude | Longitude |
| --- | --- | --- | --- | --- |
| 1 | Jaffna | Sri Lanka | 9.69 | 80.25 |
| 2 | Tamil Nadu | India | 8.66 | 80.21 |
| 3 | Jaffna | Sri Lanka | 9.69 | 80.21 |
| 4 | Matale | Sri Lanka | 7.58 | 80.24 |
| 5 | Matale | Sri Lanka | 7.55 | 79.83 |
| 6 | Kurunegala | Sri Lanka | 7.28 | 80.36 |
| 7 | Sinhala | Sri Lanka | 6.92 | 79.85 |
| 8 | Tamil Nadu | India | 10.57 | 106.65 |
| 9 | Tamil Nadu | India | 13.06 | 121.32 |
| 10 | Luzon | Philippines | 14.07 | 121.32 |
| 11 | Central Luzon | Philippines | 13.92 | 122.95 |
| 12 | Central Luzon | Philippines | 13.17 | 123.7 |
| 13 | Visayas | Philippines | 14.11 | 121.15 |
| 14 | Selangor | Malaysia | 3.73 | 101.15 |
| 15 | Central | Singapore | 1.3 | 100.23 |
| 16 | Arau | Malaysia | 2.76 | 102.28 |
| 17 | Kodiang | Malaysia | 2.22 | 102.24 |
| 18 | West Bengal | India | 22.99 | 87.01 |
| 19 | Chhattisgarh | India | 20.73 | 81.53 |
| 20 | Balod | India | 20.73 | 81.21 |
| 21 | Balrampur | India | 27.43 | 82.18 |
| 22 | Bastar | India | 19.11 | 81.95 |
| 23 | Bemetara | India | 21.71 | 81.54 |
| 24 | Bilaspur | India | 22.08 | 82.14 |
| 25 | Dantewada | India | 18.9 | 81.35 |
| 26 | Dhamtari | India | 20.71 | 81.55 |
| 27 | Durg | India | 21.19 | 81.28 |
| 28 | Gariyaband | India | 20.63 | 82.06 |
| 29 | Jashpur | India | 22.88 | 84.14 |
| 30 | West Bengal | India | 21.76 | 76.66 |
| 31 | Telangana | India | 17.47 | 80.18 |
| 32 | Telangana | India | 17.55 | 96.45 |
| 33 | West Bengal | India | 22.55 | 77.25 |
| 34 | Khon Kaen | Thailand | 16.39 | 79.92 |
| 35 | Karnataka | India | 12.38 | 79.56 |
| 36 | Tamil Nadu | India | 12.91 | 79.79 |
| 37 | Maha Sarakham | Thailand | 16.24 | 81.19 |
| 38 | Yangon | Myanmar | 16.52 | 79.99 |
| 39 | NCT of Delhi | India | 28.54 | 83.92 |
| 40 | Tamil Nadu | India | 11.1 | 76.85 |
| 41 | Gadwal | India | 16.77 | 76.19 |
| 42 | Gudur | India | 14.91 | 78.48 |
| 43 | Kerala | India | 8.6 | 77.75 |
| 44 | Kerala | India | 10.62 | 77.24 |
| 45 | Tamil Nadu | India | 13.3 | 80.25 |
| 46 | Nirmal | India | 20.26 | 76.19 |
| 47 | Telangana | India | 17.43 | 80.03 |
| 48 | Ninh BÃ¬nh | Vietnam | 20.28 | 77.45 |
| 49 | Tamil Nadu | India | 8.72 | 77.5 |
| 50 | Tamil Nadu | India | 12.95 | 79.79 |
| 51 | Kerala | India | 10.62 | 76.19 |
| 52 | Assam | India | 24.43 | 77.54 |
| 53 | Meghalaya | India | 25.35 | 79.99 |
| 54 | Yangon | Myanmar | 16.79 | 80.03 |
| 55 | Palwancha | India | 18.34 | 80.05 |
| 56 | West Bengal | India | 22.46 | 80.75 |
| 57 | West Bengal | India | 22.53 | 80.24 |
| 58 | West Bengal | India | 22.47 | 79.46 |
| 59 | West Bengal | India | 22.47 | 95.59 |
| 60 | Kerala | India | 12.24 | 79.46 |
| 61 | West Bengal | India | 22.99 | 79.8 |
| 62 | Tamil Nadu | India | 12.99 | 77.12 |
| 63 | Assam | India | 27.48 | 88.47 |
| 64 | Andhra Pradesh | India | 13.65 | 78.65 |
| 65 | Palghar | India | 19.7 | 72.77 |
| 66 | Thane | India | 19.22 | 72.98 |
| 67 | Raigad | India | 18.74 | 73.09 |
| 68 | Ratnagiri | India | 16.99 | 73.31 |
| 69 | Sindhudurg | India | 16.17 | 73.56 |
| 70 | West Bengal | India | 25.63 | 92.33 |
| 71 | Assam | India | 26.44 | 80.24 |
| 72 | Majuli | India | 27 | 94.22 |
| 73 | Dhemaji | India | 27.48 | 94.56 |
| 74 | Dibrugarh | India | 27.47 | 94.91 |
| 75 | Golaghat | India | 26.52 | 93.96 |
| 76 | Sibsagar | India | 26.98 | 94.64 |
| 77 | Uttarakhand | India | 28.95 | 87.01 |
| 78 | Delhi | India | 28.04 | 96.89 |
| 79 | West Bengal | India | 22.99 | 96.92 |
| 80 | Purba Medinipur | India | 21.94 | 87.78 |
| 81 | Bankura | India | 23.23 | 87.08 |
| 82 | Jhargram | India | 22.46 | 87 |
| 83 | Purulia | India | 23.33 | 86.36 |
| 84 | Hooghly | India | 22.9 | 88.39 |
| 85 | Telangana | India | 17.52 | 80.25 |
| 86 | Yangon | Myanmar | 16.85 | 78.11 |
| 87 | Phetchaburi | Thailand | 13.18 | 79.09 |
| 88 | Chhattisgarh | India | 19.14 | 79.86 |
| 89 | West Bengal | India | 22.44 | 95.64 |
| 90 | Tamil Nadu | India | 13.02 | 76.65 |
| 91 | Madhya Pradesh | India | 22.14 | 75.84 |
| 92 | Assam | India | 24.43 | 76.52 |
| 93 | Maharashtra | India | 19.92 | 85.94 |
| 94 | Tamil Nadu | India | 13.01 | 80.21 |
| 95 | Vientiane [prefecture] | Laos | 17.97 | 76.47 |
| 96 | West Bengal | India | 23.01 | 80.42 |
| 97 | Chittagong | Bangladesh | 22.48 | 78.34 |
| 98 | Shan | Myanmar | 20.49 | 80.41 |
| 99 | Shan | Myanmar | 20.52 | 85.83 |
| 100 | Malappuram | India | 11.05 | 76.07 |
| 101 | Kuttanad | India | 9.35 | 76.4 |
| 102 | Ernakulam | India | 9.98 | 76.3 |
| 103 | Wayanad | India | 11.69 | 76.13 |
| 104 | Palakkad | India | 10.79 | 76.65 |
| 105 | Odisha | India | 20.45 | 84.27 |
| 106 | Karnataka | India | 15.33 | 76.47 |
| 107 | Maharashtra | India | 21.17 | 80.41 |
| 108 | Maharashtra | India | 21.17 | 80.42 |
| 109 | Telangana | India | 17.45 | 78.34 |
| 110 | Maharashtra | India | 21.17 | 80.41 |
| 111 | Baidyabati | India | 20.5 | 85.83 |
| 112 | Sangarh | Pakistan | 26.03 | 68.93 |
| 113 | Jamrao Head | Pakistan | 26.42 | 68.85 |
| 114 | Sindh | Pakistan | 26.03 | 68.93 |
| 115 | Sindh | Pakistan | 26.42 | 68.85 |
| 116 | Hyderabad | India | 17.32 | 78.39 |
| 117 | Don Chedi | Thailand | 14.63 | 99.92 |
| 118 | Maharashtra | India | 19.93 | 99.87 |
| 119 | West Bengal | India | 22.99 | 99.36 |
| 120 | Chiang Mai | Thailand | 20.06 | 99.36 |
| 121 | Telangana | India | 17.49 | 103.25 |
| 122 | Telangana | India | 17.55 | 102.98 |
| 123 | West Bengal | India | 22.4 | 103.28 |
| 124 | Laos | Indonesia | 18.51 | 103.8 |
| 125 | Ha Tinh | Vietnam | 18.35 | 105.86 |
| 126 | Chiang Rai | Thailand | 20.42 | 105.86 |
| 127 | Chiang Mai | Thailand | 18.81 | 101.21 |
| 128 | Laos | Vietnam | 18.73 | 104.99 |
| 129 | Krabi | Thailand | 8.17 | 99.02 |
| 130 | West Bengal | India | 22.61 | 103.25 |
| 131 | West Bengal | India | 22.55 | 103.25 |
| 132 | West Bengal | India | 22.68 | 103.9 |
| 133 | West Bengal | India | 22.72 | 101.91 |
| 134 | Assam | India | 26.69 | 109.51 |
| 135 | Punjab | India | 30.24 | 121.15 |
| 136 | Hainan | China | 18.94 | 109.51 |
| 137 | Guangxi | China | 23.9 | 106.62 |
| 138 | Guangxi | China | 23.15 | 108.28 |
| 139 | Guizhou | China | 25.14 | 104.96 |
| 140 | Maha Sarakham | Thailand | 16.24 | 107.77 |
| 141 | Maha Sarakham | Thailand | 16.24 | 107.77 |
| 142 | Tamil Nadu | India | 9.92 | 106.62 |
| 143 | Dak Lak | Vietnam | 12.25 | 108.28 |
