# Supplementary material for: The global invasion risk of rice yellow stem borer Scirpophaga incertulas Walker (Lepidoptera:Crambidae) under current and future climate scenarios
Source: PLoS One. 2025 Mar 5;20(3):e0310234. doi: 10.1371/journal.pone.0310234 (PMC11882091; doi:10.1371/journal.pone.0310234)
Supplement: S1 File — (ZIP) [file pone.0310234.s002.zip › weather data/Faizabafd.docx]

2018

| **Week No.** | **Temperature**  **^0^C** | | **Rainfall**  **(mm.)** | **RH**  **(%)** | **VP**  **(mm)** | **Wind**  **Speed**  **(Km/hr)** | **Bright**  **Sunshine (hrs.)** | **Total**  **Evapo-**  **ration**  **(mm)** |
| --- | --- | --- | --- | --- | --- | --- | --- | --- |
|  | **Max.** | **Min.** |  |  |  |  |  |  |
| 1 | 15.1 | 4.7 | 0.0 | 84.3 | 8.4 | 2.8 | 0.6 | 22.1 |
| 2 | 13.8 | 5.2 | 0.0 | 87.8 | 8.3 | 2.3 | 0.5 | 21.0 |
| 3 | 21.8 | 4.8 | 0.0 | 74.1 | 8.3 | 2.2 | 4.1 | 29.7 |
| 4 | 21.3 | 5.9 | 1.0 | 74.8 | 8.4 | 2.7 | 4.1 | 27.6 |
| 5 | 24.3 | 7.2 | 0.0 | 70.0 | 9.8 | 2.7 | 4.7 | 30.9 |
| 6 | 24.3 | 8.1 | 0.0 | 60.7 | 8.3 | 4.1 | 4.6 | 31.0 |
| 7 | 24.7 | 10.7 | 0.0 | 72.2 | 11.4 | 4.0 | 3.5 | 35.9 |
| 8 | 28.7 | 11.8 | 0.0 | 66.3 | 12.5 | 2.2 | 6.5 | 39.6 |
| 9 | 30.1 | 14.1 | 0.0 | 67.3 | 12.3 | 4.0 | 6.6 | 40.8 |
| 10 | 31.1 | 12.3 | 0.0 | 64.7 | 11.0 | 3.9 | 6.6 | 39.8 |
| 11 | 32.8 | 13.7 | 0.0 | 58.2 | 12.6 | 3.3 | 7.1 | 42.2 |
| 12 | 34.2 | 14.2 | 0.0 | 54.5 | 11.2 | 3.6 | 7.1 | 43.0 |
| 13 | 35.2 | 15.5 | 0.0 | 54.4 | 11.8 | 7.3 | 7.3 | 45.5 |
| 14 | 35.3 | 18.7 | 0.0 | 58.0 | 16.0 | 4.8 | 7.4 | 49.6 |
| 15 | 35.4 | 18.3 | 0.0 | 53.2 | 15.0 | 3.8 | 7.1 | 49.9 |
| 16 | 39.2 | 20.5 | 0.0 | 41.9 | 12.6 | 4.2 | 7.9 | 59.9 |
| 17 | 37.6 | 19.7 | 0.0 | 46.4 | 14.1 | 6.2 | 7.8 | 53.7 |
| 18 | 35.2 | 21.8 | 8.8 | 61.6 | 19.4 | 3.3 | 7.0 | 47.9 |
| 19 | 39.5 | 23.7 | 0.0 | 46.6 | 16.7 | 4.9 | 8.9 | 61.4 |
| 20 | 36.5 | 25.2 | 11.0 | 64.6 | 22.4 | 5.4 | 8.2 | 52.2 |
| 21 | 41.2 | 26.6 | 0.0 | 54.1 | 21.9 | 4.3 | 9.3 | 58.2 |
| 22 | 36.0 | 26.4 | 0.0 | 64.1 | 22.0 | 6.7 | 6.7 | 51.2 |
| 23 | 38.6 | 27.9 | 0.0 | 59.5 | 23.8 | 6.4 | 5.6 | 53.5 |
| 24 | 40.1 | 27.1 | 0.0 | 55.9 | 22.5 | 5.5 | 6.5 | 53.5 |
| 25 | 41.4 | 27.9 | 0.0 | 53.4 | 22.9 | 5.9 | 8.1 | 61.8 |
| 26 | 36.6 | 26.7 | 12.4 | 68.7 | 23.6 | 6.2 | 4.9 | 51.0 |
| 27 | 36.2 | 27.4 | 5.5 | 65.1 | 24.5 | 4.1 | 5.1 | 36.8 |
| 28 | 34.0 | 26.3 | 89.8 | 89.8 | 25.5 | 5.9 | 3.8 | 38.9 |
| 29 | 33.2 | 26.6 | 86.2 | 77.1 | 25.1 | 5.4 | 5.9 | 40.6 |
| 30 | 30.8 | 26.0 | 142.9 | 88.4 | 25.2 | 5.4 | 0.6 | 26.9 |
| 31 | 28.3 | 25.0 | 147.8 | 92.6 | 24.5 | 2.5 | 0.3 | 21.8 |
| 32 | 32.6 | 26.5 | 9.6 | 79.6 | 25.7 | 1.9 | 5.1 | 34.4 |
| 33 | 33.0 | 26.2 | 55.4 | 78.8 | 25.3 | 1.1 | 4.9 | 37.1 |
| 34 | 32.7 | 25.5 | 123.0 | 86.0 | 26.2 | 1.5 | 3.9 | 30.9 |
| 35 | 31.8 | 26.0 | 66.0 | 87.8 | 26.3 | 2.2 | 3.7 | 26.8 |
| 36 | 31.4 | 25.7 | 48.4 | 84.6 | 25.3 | 3.2 | 3.5 | 27.2 |
| 37 | 32.4 | 25.0 | 27.2 | 80.3 | 24.4 | 2.9 | 7.8 | 39.1 |
| 38 | 32.4 | 24.0 | 1.6 | 77.7 | 23.4 | 5.0 | 7.0 | 39.6 |
| 39 | 33.4 | 23.9 | 0.0 | 75.4 | 23.3 | 2.6 | 8.4 | 42.9 |
| 40 | 34.3 | 21.6 | 0.0 | 71.4 | 21.5 | 2.0 | 9.2 | 46.1 |
| 41 | 32.6 | 21.0 | 0.0 | 72.5 | 19.6 | 2.4 | 8.3 | 39.7 |
| 42 | 33.5 | 17.5 | 0.0 | 65.5 | 16.8 | 1.9 | 8.4 | 38.2 |
| 43 | 32.1 | 15.3 | 0.0 | 70.7 | 16.6 | 1.4 | 8.4 | 35.6 |
| 44 | 31.8 | 15.5 | 0.0 | 69.61 | 15.7 | 1.1 | 8.7 | 40.1 |
| 45 | 28.7 | 12.7 | 0.0 | 68.3 | 12.1 | 1.4 | 7.4 | 33.8 |
| 46 | 28.7 | 13.0 | 0.0 | 71.2 | 13.0 | 2.2 | 7.2 | 25.3 |
| 47 | 27.8 | 11.0 | 0.0 | 67.5 | 11.0 | 1.7 | 7.0 | 22.8 |
| 48 | 26.8 | 11.0 | 0.0 | 70.7 | 11.5 | 1.3 | 6.7 | 21.0 |
| 49 | 25.2 | 8.5 | 0.0 | 71.0 | 10.0 | 1.5 | 6.3 | 19.8 |
| 50 | 24.2 | 7.5 | 0.0 | 69.0 | 9.1 | 1.8 | 6.1 | 21.1 |
| 51 | 23.2 | 5.0 | 0.0 | 71.3 | 8.4 | 2.0 | 6.7 | 20.4 |
| 52 | 21.1 | 3.5 | 0.0 | 70.8 | 6.6 | 2.4 | 7.2 | 20.9 |

2019

| **Week No.** | **Temperature**  **^0^C** | | **Rainfall**  **(mm.)** | **RH**  **(%)** | **VP**  **(mm)** | **Wind**  **Speed**  **(Km/hr)** | **Bright**  **Sunshine (hrs.)** | **Total**  **Evapo-**  **ration**  **(mm)** |
| --- | --- | --- | --- | --- | --- | --- | --- | --- |
|  | **Max.** | **Min.** |  |  |  |  |  |  |
| 1 | 22.2 | 5.3 | 0.0 | 72.2 | 8.6 | 2.6 | 5.5 | 18.7 |
| 2 | 21.8 | 5.7 | 0.0 | 72.0 | 8.5 | 2.1 | 6.0 | 21.8 |
| 3 | 22.5 | 5.0 | 0.0 | 70.5 | 8.6 | 2.5 | 6.9 | 23.2 |
| 4 | 21.1 | 10.6 | 41.0 | 76.1 | 10.9 | 4.4 | 4.2 | 26.8 |
| 5 | 21.7 | 7.0 | 0.0 | 74.9 | 9.4 | 3.4 | 7.4 | 24.4 |
| 6 | 22.3 | 8.9 | 9.0 | 75.1 | 10.6 | 3.6 | 4.3 | 24.4 |
| 7 | 21.8 | 10.5 | 0.0 | 76.6 | 11.2 | 3.2 | 3.6 | 23.0 |
| 8 | 25.3 | 11.2 | 0.0 | 69.1 | 17.8 | 3.8 | 6.9 | 31.8 |
| 9 | 22.9 | 10.0 | 0.0 | 72.7 | 11.1 | 3.6 | 6.7 | 28.8 |
| 10 | 26.5 | 10.6 | 0.0 | 66.9 | 12.2 | 3.7 | 9.2 | 33.2 |
| 11 | 30.0 | 12.7 | 0.0 | 61.4 | 11.4 | 2.8 | 7.6 | 36.2 |
| 12 | 31.9 | 14.2 | 0.0 | 60.5 | 12.4 | 4.6 | 9.4 | 38.6 |
| 13 | 34.0 | 17.4 | 0.0 | 62.1 | 15.2 | 2.6 | 8.8 | 40.5 |
| 14 | 34.8 | 18.9 | 2.0 | 61.3 | 16.2 | 4.3 | 8.4 | 44.4 |
| 15 | 37.5 | 21.2 | 0.0 | 56.6 | 18.3 | 3.3 | 9.4 | 50.5 |
| 16 | 35.0 | 19.2 | 0.0 | 66.6 | 19.2 | 4.8 | 7.9 | 42.4 |
| 17 | 37.2 | 23.2 | 0.0 | 53.0 | 18.2 | 5.1 | 9.5 | 50.2 |
| 18 | 40.0 | 24.5 | 0.0 | 54.6 | 19.3 | 6.3 | 9.9 | 50.8 |
| 19 | 42.4 | 24.9 | 0.0 | 45.5 | 18.4 | 8.3 | 8.7 | 56.8 |
| 20 | 39.5 | 24.2 | 0.0 | 45.8 | 18.3 | 4.8 | 9.4 | 56.2 |
| 21 | 41.3 | 25.7 | 0.0 | 44.3 | 17.1 | 5.4 | 9.4 | 63.0 |
| 22 | 40.4 | 24.9 | 0.0 | 46.8 | 10.7 | 6.9 | 9.8 | 61.8 |
| 23 | 39.9 | 26.5 | 0.0 | 52.6 | 21.2 | 6.2 | 8.7 | 61.6 |
| 24 | 41.2 | 27.9 | 0.0 | 49.1 | 21.0 | 7.5 | 8.3 | 61.0 |
| 25 | 36.4 | 25.6 | 24.0 | 61.5 | 22.0 | 9.5 | 5.6 | 49.5 |
| 26 | 38.0 | 27.8 | 0.0 | 60.3 | 23.6 | 5.1 | 6.9 | 49.5 |
| 27 | 35.9 | 27.5 | 25.8 | 74.8 | 25.1 | 6.9 | 5.6 | 52.1 |
| 28 | 31.2 | 24.9 | 250.9 | 84.6 | 25.1 | 6.7 | 0.5 | 33.2 |
| 29 | 35.2 | 26.1 | 18.4 | 84.4 | 27.2 | 3.0 | 5.8 | 44.6 |
| 30 | 31.9 | 25.6 | 101.4 | 82.9 | 24.5 | 4.9 | 3.5 | 32.6 |
| 31 | 33.4 | 27.0 | 16.6 | 78.8 | 26.0 | 5.8 | 6.2 | 40.2 |
| 32 | 33.8 | 26.6 | 18.0 | 78.4 | 26.0 | 5.8 | 6.2 | 42.9 |
| 33 | 32.7 | 26.7 | 4.0 | 86.2 | 25.9 | 5.0 | 6.4 | 44.2 |
| 34 | 31.1 | 24.7 | 84.6 | 86.2 | 25.7 | 3.0 | 1.6 | 36.4 |
| 35 | 34.0 | 26.0 | 0.0 | 79.9 | 27.1 | 3.1 | 6.4 | 45.0 |
| 36 | 33.7 | 26.9 | 10.0 | 81.6 | 27.2 | 3.9 | 7.2 | 41.2 |
| 37 | 32.7 | 26.5 | 104.0 | 86.8 | 26.5 | 4.2 | 3.7 | 35.4 |
| 38 | 30.0 | 24.2 | 103.0 | 89.1 | 24.4 | 2.6 | 2.0 | 32.2 |
| 39 | 27.5 | 22.9 | 179.0 | 91.7 | 22.3 | 5.7 | 0.7 | 26.4 |
| 40 | 30.3 | 22.4 | 10.0 | 82.5 | 22.5 | 1.6 | 4.1 | 31.1 |
| 41 | 32.5 | 20.9 | 0.0 | 74.1 | 19.8 | 1.2 | 7.8 | 32.0 |
| 42 | 31.8 | 21.0 | 0.0 | 77.9 | 19.9 | 0.8 | 6.1 | 35.0 |
| 43 | 29.2 | 17.4 | 0.0 | 75.9 | 16.3 | 2.0 | 4.9 | 28.4 |
| 44 | 29.2 | 16.9 | 0.0 | 78.8 | 17.4 | 0.8 | 2.0 | 23.7 |
| 45 | 29.7 | 15.5 | 0.0 | 74.0 | 15.4 | 1.9 | 4.0 | 23.4 |
| 46 | 29.4 | 13.1 | 0.0 | 81.6 | 16.8 | 1.9 | 6.8 | 23.6 |
| 47 | 27.2 | 12.5 | 0.0 | 75.4 | 13.3 | 2.2 | 6.2 | 22.2 |
| 48 | 26.8 | 14.2 | 0.0 | 77.5 | 13.9 | 1.6 | 3.5 | 22.5 |
| 49 | 24.9 | 9.7 | 0.0 | 70.7 | 10.8 | 1.7 | 6.2 | 21.9 |
| 50 | 23.1 | 11.2 | 22.0 | 74.4 | 11.0 | 3.4 | 4.1 | 20.8 |
| 51 | 17.6 | 8.5 | 0.0 | 82.7 | 9.7 | 3.9 | 2.6 | 18.6 |
| 52 | 14.0 | 5.3 | 0.0 | 84.7 | 7.2 | 3.2 | 0.9 | 15.5 |

2020

| **Week No.** | **Temperature**  **^0^C** | | **Rainfall**  **(mm.)** | **RH**  **(%)** | **VP**  **(mm)** | **Wind**  **Speed**  **(Km/hr)** | **Bright**  **Sunshine (hrs.)** | **Total**  **Evapo-**  **ration**  **(mm)** |
| --- | --- | --- | --- | --- | --- | --- | --- | --- |
|  | **Max.** | **Min.** |  |  |  |  |  |  |
| 1 | 19.3 | 9.1 | 7.8 | 75.7 | 9.9 | 3.3 | 3.8 | 15.3 |
| 2 | 16.5 | 8.2 | 6.6 | 85.4 | 9.4 | 3.5 | 1.9 | 14.2 |
| 3 | 18.6 | 10.2 | 21.0 | 86.1 | 10.9 | 3.2 | 1.5 | 17.0 |
| 4 | 20.5 | 6.4 | 0.0 | 78.2 | 10.0 | 3.5 | 6.5 | 21.3 |
| 5 | 21.8 | 7.3 | 0.0 | 74.2 | 9.8 | 4.4 | 6.7 | 24.4 |
| 6 | 22.5 | 6.2 | 0.0 | 70.3 | 9.0 | 6.5 | 8.1 | 23.2 |
| 7 | 22.8 | 8.8 | 0.0 | 67.3 | 10.4 | 5.4 | 8.4 | 25.8 |
| 8 | 25.4 | 12.4 | 53.0 | 76.5 | 13.6 | 4.3 | 4.7 | 27.0 |
| 9 | 26.8 | 14.0 | 0.0 | 72.6 | 14.4 | 2.1 | 7.5 | 25.2 |
| 10 | 26.2 | 13.9 | 68.0 | 73.9 | 14.3 | 5.3 | 6.4 | 26.2 |
| 11 | 26.8 | 14.7 | 9.0 | 73.3 | 14.3 | 3.4 | 5.7 | 26.6 |
| 12 | 29.5 | 15.7 | 2.0 | 68.4 | 15.3 | 2.6 | 8.0 | 34.4 |
| 13 | 31.9 | 16.1 | 0.0 | 54.2 | 12.9 | 6.4 | 8.9 | 37.0 |
| 14 | 34.7 | 16.0 | 0.0 | 46.0 | 11.1 | 4.9 | 9.9 | 45.0 |
| 15 | 36.6 | 18.7 | 0.0 | 50.4 | 14.8 | 3.3 | 9.2 | 45.8 |
| 16 | 36.8 | 21.5 | 1.0 | 55.9 | 16.8 | 4.2 | 9.3 | 45.8 |
| 17 | 33.0 | 22.0 | 11.0 | 64.5 | 17.9 | 5.0 | 8.7 | 38.6 |
| 18 | 32.6 | 21.1 | 27.8 | 69.2 | 18.6 | 4.8 | 9.1 | 37.8 |
| 19 | 33.7 | 22.1 | 10.0 | 62.4 | 19.2 | 4.4 | 9.5 | 42.0 |
| 20 | 39.0 | 22.7 | 0.0 | 49.1 | 16.5 | 4.2 | 10.2 | 44.8 |
| 21 | 40.0 | 25.1 | 0.0 | 52.0 | 20.0 | 4.4 | 9.7 | 45.6 |
| 22 | 35.5 | 25.0 | 12.2 | 63.6 | 20.8 | 6.6 | 6.9 | 45.0 |
| 23 | 34.1 | 25.5 | 9.2 | 70.4 | 22.2 | 3.9 | 5.3 | 38.4 |
| 24 | 36.1 | 28.5 | 47.8 | 69.4 | 25.0 | 6.8 | 8.0 | 45.0 |
| 25 | 31.6 | 26.2 | 96.2 | 84.9 | 25.3 | 5.0 | 1.7 | 34.0 |
| 26 | 31.5 | 25.6 | 77.6 | 86.7 | 24.8 | 4.1 | 1.5 | 35.4 |
| 27 | 32.6 | 26.8 | 105.0 | 84.9 | 26.5 | 4.5 | 1.9 | 32.2 |
| 28 | 32.8 | 27.2 | 57.2 | 81.2 | 25.3 | 4.8 | 2.2 | 33.8 |
| 29 | 33.0 | 27.2 | 20.2 | 80.9 | 26.1 | 4.2 | 3.5 | 34.0 |
| 30 | 33.4 | 26.5 | 66.4 | 83.8 | 25.8 | 3.7 | 3.5 | 32.8 |
| 31 | 33.1 | 26.5 | 50.2 | 83.7 | 22.8 | 2.4 | 1.7 | 32.6 |
| 32 | 33.7 | 27.3 | 73.0 | 81.5 | 25.7 | 5.3 | 4.5 | 34.2 |
| 33 | 32.6 | 27.3 | 114.8 | 85.3 | 26.6 | 3.7 | 3.5 | 30.8 |
| 34 | 32.2 | 26.2 | 37.4 | 78.7 | 24.6 | 5.9 | 3.2 | 33.4 |
| 35 | 32.6 | 26.0 | 6.0 | 76.9 | 24.0 | 5.1 | 6.3 | 32.8 |
| 36 | 34.2 | 26.3 | 16.2 | 79.9 | 25.9 | 2.5 | 5.6 | 33.4 |
| 37 | 34.6 | 26.8 | 0.0 | 73.4 | 24.3 | 1.9 | 3.5 | 35.4 |
| 38 | 34.0 | 26.5 | 0.0 | 78.2 | 24.7 | 3.1 | 6.3 | 34.0 |
| 39 | 31.9 | 24.0 | 76.6 | 76.9 | 22.8 | 2.9 | 4.6 | 29.2 |
| 40 | 34.4 | 23.0 | 3.0 | 73.6 | 21.7 | 1.1 | 6.7 | 32.4 |
| 41 | 34.3 | 21.4 | 0.0 | 70.3 | 19.9 | 1.2 | 7.8 | 31.0 |
| 42 | 34.2 | 21.1 | 0.0 | 68.1 | 18.3 | 1.2 | 7.0 | 30.6 |
| 43 | 32.9 | 16.4 | 0.0 | 63.6 | 14.6 | 1.8 | 7.6 | 28.6 |
| 44 | 31.3 | 13.5 | 0.0 | 60.7 | 11.6 | 1.9 | 7.7 | 29.2 |
| 45 | 30.0 | 11.7 | 0.0 | 62.6 | 10.7 | 0.8 | 5.1 | 29.6 |
| 46 | 28.1 | 14.8 | 6.2 | 71.1 | 13.7 | 1.5 | 6.7 | 30.4 |
| 47 | 24.5 | 10.2 | 0.0 | 64.0 | 9.4 | 1.7 | 7.2 | 30.0 |
| 48 | 26.8 | 8.8 | 0.0 | 64.1 | 9.1 | 1.4 | 7.5 | 30.6 |
| 49 | 27.3 | 10.2 | 0.0 | 70.4 | 9.2 | 0.7 | 5.2 | 28.8 |
| 50 | 19.8 | 11.0 | 0.0 | 74.2 | 11.3 | 1.5 | 1.9 | 26.8 |
| 51 | 10.1 | 5.1 | 0.0 | 68.0 | 7.7 | 2.2 | 6.0 | 26.2 |
| 52 | 26.0 | 5.9 | 0.0 | 76.4 | 9.5 | 2.0 | 8.3 | 27.0 |

2021

| **Week No.** | **Temperature**  **^0^C** | | **Rainfall**  **(mm.)** | **RH**  **(%)** | **VP**  **(mm)** | **Wind**  **Speed**  **(Km/hr)** | **Bright**  **Sunshine (hrs.)** | **Total**  **Evapo-**  **ration**  **(mm)** |
| --- | --- | --- | --- | --- | --- | --- | --- | --- |
|  | **Max.** | **Min.** |  |  |  |  |  |  |
| 1 | 24.2 | 9.5 | 0.0 | 66.4 | 10.1 | 5.2 | 4.9 | 26.6 |
| 2 | 21.3 | 8.8 | 0.0 | 74.1 | 9.5 | 3.9 | 5.8 | 26.0 |
| 3 | 18.5 | 7.4 | 0.0 | 80.2 | 9.3 | 1.8 | 2.8 | 17.4 |
| 4 | 16.8 | 7.0 | 0.0 | 84.4 | 9.1 | 1.3 | 3.7 | 12.6 |
| 5 | 21.1 | 5.5 | 0.0 | 71.1 | 8.8 | 1.4 | 4.5 | 16.8 |
| 6 | 25.5 | 9.0 | 0.0 | 67.1 | 10.2 | 2.2 | 6.3 | 25.8 |
| 7 | 27.2 | 9.5 | 0.0 | 68.1 | 10.6 | 1.4 | 6.8 | 29.2 |
| 8 | 28.2 | 11.7 | 0.0 | 66.3 | 11.6 | 1.8 | 7.6 | 31.0 |
| 9 | 30.2 | 13.2 | 0.0 | 58.8 | 12.2 | 4.8 | 7.0 | 33.8 |
| 10 | 31.8 | 14.2 | 0.0 | 64.3 | 15.5 | 2.9 | 6.6 | 36.9 |
| 11 | 32.3 | 15.2 | 0.0 | 66.4 | 15.5 | 2.9 | 6.2 | 37.6 |
| 12 | 34.6 | 16.5 | 0.0 | 57.0 | 14.6 | 2.7 | 6.8 | 41.6 |
| 13 | 35.6 | 16.4 | 0.0 | 56.3 | 13.8 | 3.9 | 7.5 | 40.6 |
| 14 | 37.5 | 17.0 | 0.0 | 44.3 | 14.1 | 3.4 | 6.7 | 41.8 |
| 15 | 33.3 | 18.8 | 0.0 | 34.5 | 10.7 | 3.5 | 6.2 | 42.4 |
| 16 | 37.9 | 20.1 | 0.0 | 45.6 | 14.2 | 4.0 | 7.5 | 41.0 |
| 17 | 38.5 | 17.7 | 0.0 | 58.6 | 16.8 | 3.4 | 7.5 | 41.4 |
| 18 | 36.6 | 24.2 | 0.0 | 51.6 | 16.5 | 4.2 | 6.9 | 41.6 |
| 19 | 35.2 | 23.9 | 30.2 | 58.7 | 17.8 | 5.4 | 6.7 | 41.6 |
| 20 | 33.3 | 24.0 | 34.6 | 71.6 | 20.1 | 3.3 | 4.3 | 38.4 |
| 21 | 34.0 | 24.3 | 16.2 | 64.7 | 20.4 | 5.8 | 6.7 | 41.4 |
| 22 | 31.6 | 24.3 | 92.8 | 78.6 | 23.0 | 4.3 | 5.8 | 39.2 |
| 23 | 35.9 | 27.0 | 2.8 | 66.2 | 22.6 | 2.7 | 8.0 | 42.4 |
| 24 | 30.2 | 25.4 | 101.8 | 85.8 | 25.2 | 3.4 | 2.8 | 37.8 |
| 25 | 31.5 | 25.7 | 62.6 | 73.1 | 24.5 | 2.4 | 3.0 | 37.8 |
| 26 | 34.1 | 27.5 | 14.4 | 77.7 | 25.9 | 1.9 | 4.0 | 40.0 |
| 27 | 35.5 | 27.2 | 33.4 | 83.6 | 27.6 | 3.2 | 5.7 | 43.0 |
| 28 | 34.8 | 27.1 | 0.0 | 74.3 | 25.9 | 3.4 | 6.7 | 44.8 |
| 29 | 34.1 | 26.8 | 38.0 | 80.9 | 26.2 | 3.8 | 3.0 | 44.4 |
| 30 | 34.4 | 27.2 | 37.4 | 79.6 | 26.3 | 2.8 | 4.2 | 43.2 |
| 31 | 33.2 | 26.1 | 3.0 | 76.0 | 24.4 | 3.1 | 5.9 | 41.6 |
| 32 | 32.2 | 26.2 | 125.6 | 85.9 | 25.6 | 1.9 | 3.4 | 41.6 |
| 33 | 33.8 | 26.2 | 18.8 | 80.8 | 26.2 | 2.9 | 5.4 | 43.2 |
| 34 | 32.2 | 25.5 | 24.6 | 87.8 | 25.4 | 2.3 | 2.6 | 41.2 |
| 35 | 32.7 | 26.1 | 1.0 | 81.2 | 25.2 | 2.3 | 5.5 | 42.8 |
| 36 | 35.7 | 26.2 | 42.4 | 75.4 | 25.6 | 4.6 | 3.3 | 44.4 |
| 37 | 32.1 | 25.5 | 206.0 | 78.8 | 23.8 | 4.0 | 4.4 | 42.0 |
| 38 | 31.2 | 25.0 | 41.4 | 85.8 | 25.4 | 7.5 | 6.1 | 38.2 |
| 39 | 32.2 | 25.1 | 63.0 | 84.5 | 25.4 | 1.4 | 7.3 | 41.6 |
| 40 | 32.7 | 24.5 | 18.0 | 82.8 | 25.4 | 4.0 | 8.1 | 43.4 |
| 41 | 34.0 | 24.0 | 0.0 | 79.5 | 24.9 | 1.8 | 9.0 | 44.4 |
| 42 | 31.8 | 23.6 | 24.0 | 81.3 | 22.2 | 5.2 | 6.9 | 39.8 |
| 43 | 31.1 | 18.2 | 0.0 | 68.0 | 14.2 | 1.2 | 6.5 | 29.0 |
| 44 | 29.7 | 15.0 | 0.0 | 68.3 | 13.9 | 1.8 | 6.6 | 23.1 |
| 45 | 29.7 | 13.7 | 0.0 | 69.4 | 13.9 | 2.1 | 6.5 | 22.6 |
| 46 | 27.7 | 12.3 | 0.0 | 77.3 | 14.7 | 1.5 | 7.0 | 22.5 |
| 47 | 27.4 | 13.5 | 0.0 | 76.0 | 14.7 | 1.6 | 3.6 | 19.8 |
| 48 | 27.5 | 11.4 | 0.0 | 79.8 | 13.8 | 2.1 | 5.9 | 18.4 |
| 49 | 27.5 | 12.6 | 0.0 | 74.7 | 13.3 | 7.5 | 4.7 | 21.4 |
| 50 | 22.8 | 7.9 | 0.0 | 79.4 | 10.9 | 3.2 | 6.2 | 19.4 |
| 51 | 21.7 | 5.8 | 0.0 | 77.0 | 9.6 | 2.4 | 5.0 | 19.6 |
| 52 | 21.3 | 9.9 | 15.0 | 81.5 | 10.8 | 2.0 | 2.5 | 16.9 |

2022

| **Week No.** | **Temperature**  **^0^C** | | **Rainfall**  **(mm.)** | **RH**  **(%)** | **VP**  **(mm)** | **Wind**  **Speed**  **(Km/hr)** | **Bright**  **Sunshine (hrs.)** | **Total**  **Evapo-**  **ration**  **(mm)** |
| --- | --- | --- | --- | --- | --- | --- | --- | --- |
|  | **Max.** | **Min.** |  |  |  |  |  |  |
| 1 | 19.4 | 9.3 | 14.2 | 82.5 | 10.2 | 2.4 | 2.5 | 15.4 |
| 2 | 20.2 | 10.6 | 11.6 | 87.2 | 12.0 | 2.9 | 2.1 | 16.6 |
| 3 | 15.9 | 5.7 | 0.0 | 86.6 | 9.1 | 3.2 | 1.9 | 16.6 |
| 4 | 17.3 | 8.3 | 8.8 | 87.4 | 10.0 | 3.2 | 2.9 | 15.8 |
| 5 | 19.6 | 8.8 | 3.0 | 86.7 | 10.9 | 3.7 | 4.9 | 18.2 |
| 6 | 18.4 | 8.4 | 0.0 | 84.9 | 11.2 | 3.3 | 6.0 | 19.4 |
| 7 | 24.0 | 9.9 | 0.0 | 80.4 | 12.5 | 3.9 | 8.3 | 25.6 |
| 8 | 25.6 | 13.5 | 0.0 | 77.7 | 12.9 | 5.1 | 8.1 | 34.4 |
| 9 | 27.1 | 11.4 | 0.0 | 79.6 | 14.2 | 5.5 | 9.0 | 36.4 |
| 10 | 29.1 | 12.5 | 0.0 | 77.3 | 15.1 | 4.2 | 6.9 | 40.0 |
| 11 | 32.0 | 16.2 | 0.0 | 77.1 | 18.8 | 4.9 | 8.3 | 45.0 |
| 12 | 35.3 | 18.5 | 0.0 | 72.9 | 20.6 | 3.9 | 8.6 | 48.6 |
| 13 | 36.8 | 16.7 | 0.0 | 58.9 | 17.5 | 4.0 | 8.3 | 48.8 |
| 14 | 36.3 | 17.2 | 0.0 | 56.9 | 18.8 | 4.3 | 8.3 | 44.2 |
| 15 | 39.2 | 19.6 | 0.0 | 54.1 | 18.6 | 5.2 | 8.3 | 34.8 |
| 16 | 40.7 | 22.4 | 0.0 | 53.1 | 21.3 | 4.0 | 9.4 | 41.8 |
| 17 | 38.2 | 22.2 | 0.0 | 51.5 | 20.8 | 5.4 | 9.0 | 41.0 |
| 18 | 37.0 | 24.0 | 19.2 | 61.9 | 21.8 | 6.3 | 8.7 | 37.2 |
| 19 | 36.3 | 25.7 | 0.0 | 64.7 | 24.1 | 4.3 | 8.1 | 40.6 |
| 20 | 40.1 | 26.9 | 0.0 | 63.6 | 25.2 | 4.5 | 8.2 | 42.4 |
| 21 | 35.6 | 23.3 | 19.2 | 65.1 | 23.2 | 5.1 | 6.1 | 33.8 |
| 22 | 39.0 | 26.0 | 2.0 | 59.9 | 23.8 | 4.1 | 8.3 | 45.2 |
| 23 | 41.8 | 26.9 | 0.0 | 56.6 | 24.7 | 4.6 | 9.3 | 56.0 |
| 24 | 41.9 | 27.9 | 0.0 | 57.4 | 25.3 | 6.1 | 9.1 | 51.8 |
| 25 | 37.3 | 26.7 | 2.2 | 62.1 | 24.7 | 5.3 | 6.5 | 45.8 |
| 26 | 34.0 | 26.2 | 51.8 | 68.5 | 23.5 | 5.9 | 4.0 | 43.2 |
| 27 | 35.4 | 27.5 | 3.0 | 66.7 | 25.9 | 5.0 | 6.5 | 38.9 |
| 28 | 36.7 | 27.4 | 3.0 | 64.1 | 25.8 | 7.7 | 9.0 | 46.4 |
| 29 | 35.1 | 27.0 | 30.8 | 71.8 | 25.4 | 6.9 | 5.5 | 40.1 |
| 30 | 32.4 | 25.1 | 83.6 | 83.6 | 26.2 | 6.3 | 1.4 | 37.2 |
| 31 | 32.8 | 25.7 | 21.2 | 83.0 | 26.7 | 4.2 | 2.5 | 30.8 |
| 32 | 33.6 | 26.2 | 8.0 | 81.5 | 26.8 | 6.6 | 7.5 | 31.0 |
| 33 | 32.5 | 25.4 | 31.8 | 84.4 | 26.8 | 7.2 | 5.7 | 32.0 |
| 34 | 32.2 | 25.5 | 65.2 | 83.0 | 26.5 | 4.6 | 7.6 | 31.0 |
| 35 | 33.7 | 25.5 | 18.8 | 78.4 | 26.7 | 2.2 | 4.0 | 34.0 |
| 36 | 34.8 | 24.7 | 11.4 | 74.1 | 25.7 | 3.0 | 7.9 | 37.6 |
| 37 | 31.5 | 24.4 | 98.6 | 85.4 | 25.0 | 7.5 | 5.0 | 32.2 |
| 38 | 31.9 | 24.7 | 70.8 | 83.4 | 25.8 | 3.5 | 4.4 | 33.5 |
| 39 | 32.5 | 23.9 | 21.2 | 84.2 | 25.2 | 1.8 | 5.0 | 33.6 |
| 40 | 31.2 | 23.0 | 163.0 | 82.2 | 23.6 | 3.0 | 3.9 | 33.7 |
| 41 | 30.6 | 21.7 | 49.0 | 83.9 | 24.2 | 2.5 | 4.0 | 25.8 |
| 42 | 30.7 | 17.9 | 0.0 | 76.5 | 20.6 | 1.1 | 8.2 | 29.2 |
| 43 | 30.9 | 15.7 | 0.0 | 73.1 | 18.2 | 1.4 | 8.5 | 27.2 |
| 44 | 30.3 | 14.8 | 0.0 | 74.3 | 18.1 | 1.2 | 7.3 | 27.2 |
| 45 | 29.5 | 16.3 | 0.0 | 74.3 | 16.8 | 1.2 | 4.7 | 27.0 |
| 46 | 28.1 | 11.3 | 0.0 | 71.6 | 13.3 | 2.8 | 7.1 | 27.8 |
| 47 | 26.7 | 9.7 | 0.0 | 66.7 | 11.6 | 1.8 | 0.0 | 25.8 |
| 48 | 26.8 | 10.2 | 0.0 | 65.6 | 11.5 | 1.0 | 6.1 | 26.1 |
| 49 | 25.4 | 8.2 | 0.0 | 71.2 | 11.4 | 1.6 | 6.4 | 23.8 |
| 50 | 25.8 | 8.3 | 0.0 | 66.0 | 10.7 | 3.2 | 8.0 | 27.6 |
| 51 | 23.5 | 7.0 | 0.0 | 69.7 | 10.1 | 2.2 | 5.2 | 26.0 |
| 52 | 19.2 | 7.1 | 0.0 | 74.3 | 9.5 | 2.3 | 3.8 | 27.6 |

2023
