# Supplementary material for: The global invasion risk of rice yellow stem borer Scirpophaga incertulas Walker (Lepidoptera:Crambidae) under current and future climate scenarios
Source: PLoS One. 2025 Mar 5;20(3):e0310234. doi: 10.1371/journal.pone.0310234 (PMC11882091; doi:10.1371/journal.pone.0310234)
Supplement: S1 File — (ZIP) [file pone.0310234.s002.zip › weather data/Hisa weather data.docx]

Hisar 2018

| **SMWs** | **T_max_**  **(°C)** | **T_min_**  **(°C)** | **G min**  **(°C)** | **AVP_m_**  **(kP)** | **AVP_e_**  **(kP)** | **RH_m_**  **(%)** | **RH_e_**  **(%)** | **WS**  **(km/hr)** | **BSS**  **(h)** | **PE**  **(mm)** | **RF**  **(mm)** | **RD**  **days** |
| --- | --- | --- | --- | --- | --- | --- | --- | --- | --- | --- | --- | --- |
| **1** | 17.5 | 4.0 | -0.5 | 6.8 | 7.8 | 100.0 | 55.9 | 1.3 | 3.8 | 0.7 | 0.0 | 0 |
| **2** | 21.9 | 2.6 | -2.5 | 6.2 | 8.6 | 94.4 | 42.7 | 1.9 | 6.9 | 1.4 | 0.0 | 0 |
| **3** | 22.9 | 5.0 | -0.6 | 7.1 | 9.8 | 93.9 | 48.3 | 2.9 | 6.9 | 1.4 | 0.0 | 0 |
| **4** | 18.2 | 7.2 | 3.7 | 8.7 | 10.3 | 96.9 | 75.3 | 2.8 | 3.8 | 1.5 | 10.9 | 1 |
| **5** | 22.9 | 5.7 | 2.3 | 8.1 | 12.8 | 97.1 | 60.1 | 1.4 | 6.9 | 1.4 | 0.0 | 0 |
| **6** | 22.0 | 4.7 | -0.1 | 7.2 | 12.9 | 86.0 | 66.7 | 1.8 | 7.0 | 1.8 | 0.0 | 0 |
| **7** | 22.8 | 8.1 | 3.3 | 9.4 | 11.0 | 93.1 | 53.6 | 3.8 | 5.7 | 2.0 | 1.2 | 0 |
| **8** | 27.9 | 10.6 | 6.1 | 11.4 | 11.9 | 90.1 | 43.7 | 1.9 | 6.5 | 2.1 | 0.0 | 0 |
| **9** | 28.1 | 12.1 | 8.5 | 13.0 | 13.6 | 91.4 | 47.1 | 2.5 | 5.9 | 2.7 | 0.0 | 0 |
| **10** | 28.9 | 10.5 | - | 12.4 | 10.8 | 86.0 | 36.3 | 2.3 | 7.2 | 3.0 | 0.0 | 0 |
| **11** | 31.8 | 12.7 | - | 11.8 | 10.8 | 80.3 | 31.3 | 2.5 | 7.3 | 3.8 | 0.0 | 0 |
| **12** | 29.8 | 12.5 | - | 13.5 | 12.9 | 81.9 | 42.3 | 3.1 | 6.7 | 3.5 | 0.0 | 0 |
| **13** | 34.7 | 13.8 | - | 12.6 | 11.2 | 73.0 | 27.3 | 3.6 | 7.8 | 5.2 | 0.0 | 0 |
| **14** | 35.7 | 19.8 | - | 14.7 | 14.7 | 61.4 | 33.4 | 5.6 | 5.4 | 5.6 | 0.0 | 0 |
| **15** | 33.7 | 18.0 | - | 15.0 | 13.9 | 72.0 | 39.1 | 6.1 | 7.0 | 5.4 | 14.0 | 1 |
| **16** | 37.6 | 19.4 | - | 13.1 | 14.3 | 56.3 | 31.0 | 5.5 | 8.3 | 7.7 | 0.0 | 0 |
| **17** | 39.3 | 20.6 | - | 14.1 | 15.0 | 52.1 | 27.0 | 5.3 | 8.5 | 6.9 | 0.0 | 0 |
| **18** | 39.4 | 25.2 | - | 16.0 | 15.6 | 56.7 | 30.1 | 9.1 | 6.8 | 9.1 | 0.0 | 0 |
| **19** | 39.1 | 22.3 | - | 15.5 | 12.7 | 56.9 | 23.9 | 7.1 | 7.5 | 7.7 | 0.0 | 0 |
| **20** | 39.7 | 23.3 | - | 17.5 | 21.3 | 62.6 | 39.9 | 6.9 | 4.7 | 8.2 | 0.0 | 0 |
| **21** | 42.5 | 22.8 | - | 14.6 | 13.2 | 47.9 | 20.6 | 4.7 | 8.6 | 9.0 | 0.0 | 0 |
| **22** | 43.8 | 26.4 | - | 22.1 | 23.2 | 66.4 | 35.7 | 8.1 | 6.6 | 9.3 | 0.0 | 0 |
| **23** | 41.7 | 28.6 | - | 26.5 | 24.2 | 77.1 | 43.1 | 9.5 | 5.6 | 8.6 | 2.2 | 0 |
| **24** | 39.7 | 29.6 | - | 23.5 | 24.2 | 67.9 | 46.0 | 12.2 | 1.6 | 11.1 | 0.0 | 0 |
| **25** | 39.4 | 26.4 | - | 20.5 | 19.2 | 61.1 | 34.7 | 5.6 | 6.7 | 9.0 | 0.0 | 0 |
| **26** | 35.1 | 26.5 | - | 24.2 | 25.2 | 81.0 | 71.3 | 7.1 | 4.1 | 5.7 | 56.7 | 2 |
| **27** | 35.7 | 26.4 | - | 24.8 | 23.9 | 81.1 | 55.3 | 6.3 | 6.5 | 5.0 | 39.1 | 1 |
| **28** | 37.2 | 28.0 | - | 26.7 | 26.9 | 84.9 | 64.3 | 6.6 | 5.8 | 5.1 | 14.2 | 1 |
| **29** | 34.0 | 26.1 | - | 26.7 | 27.6 | 94.0 | 86.4 | 4.9 | 3.9 | 3.3 | 34.4 | 3 |
| **30** | 33.2 | 26.4 | - | 26.9 | 27.9 | 95.0 | 73.7 | 4.6 | 3.0 | 2.6 | 70.8 | 2 |
| **31** | 36.4 | 26.6 | - | 23.2 | 24.2 | 78.4 | 53.3 | 7.0 | 5.8 | 6.0 | 0.0 | 0 |
| **32** | 32.9 | 26.3 | - | 26.3 | 25.9 | 91.7 | 75.4 | 6.4 | 3.5 | 3.3 | 22.1 | 1 |
| **33** | 35.6 | 27.0 | - | 27.2 | 26.8 | 88.9 | 62.4 | 6.2 | 6.6 | 4.9 | 0.6 | 0 |
| **34** | 36.0 | 27.0 | - | 26.8 | 27.9 | 89.7 | 65.4 | 7.2 | 4.9 | 4.6 | 0.8 | 0 |
| **35** | 35.5 | 26.5 | - | 25.6 | 26.8 | 88.7 | 65.7 | 5.7 | 5.1 | 4.5 | 1.1 | 0 |
| **36** | 33.5 | 25.6 | - | 24.7 | 25.7 | 89.4 | 68.6 | 5.6 | 3.0 | 3.9 | 8.9 | 1 |
| **37** | 34.4 | 23.5 | - | 23.3 | 25.6 | 90.1 | 63.6 | 6.9 | 7.6 | 5.2 | 29.2 | 1 |
| **38** | 33.7 | 23.3 | - | 22.9 | 28.1 | 93.0 | 75.6 | 5.9 | 6.6 | 4.9 | 44.6 | 2 |
| **39** | 30.8 | 21.6 | - | 21.8 | 22.5 | 97.6 | 68.7 | 3.6 | 5.1 | 2.6 | 32.0 | 1 |
| **40** | 34.4 | 20.0 | - | 18.8 | 16.3 | 89.6 | 40.0 | 3.3 | 7.9 | 4.2 | 0.0 | 0 |
| **41** | 32.5 | 15.2 | - | 15.3 | 15.2 | 86.0 | 42.6 | 4.6 | 6.8 | 3.7 | 0.0 | 0 |
| **42** | 33.4 | 16.5 | - | 14.0 | 11.3 | 72.3 | 30.1 | 3.4 | 7.1 | 3.7 | 0.0 | 0 |
| **43** | 31.4 | 14.4 | - | 12.8 | 12.1 | 84.1 | 36.1 | 2.4 | 7.1 | 2.9 | 0.0 | 0 |
| **44** | 31.0 | 15.4 | - | 14.1 | 13.9 | 91.6 | 43.7 | 2.9 | 2.1 | 2.4 | 0.0 | 0 |
| **45** | 27.4 | 10.1 | - | 10.5 | 11.6 | 89.7 | 41.1 | 2.5 | 3.3 | 2.1 | 0.0 | 0 |
| **46** | 27.5 | 12.7 | - | 12.1 | 14.1 | 91.0 | 53.1 | 3.1 | 3.5 | 1.9 | 0.0 | 0 |
| **47** | 27.4 | 10.9 | - | 10.2 | 11.9 | 87.4 | 44.2 | 3.4 | 5.8 | 2.3 | 0.0 | 0 |
| **48** | 27.2 | 9.4 | 0.0 | 9.9 | 12.1 | 93.1 | 46.3 | 1.9 | 5.5 | 1.7 | 0.0 | 0 |
| **49** | 24.9 | 7.5 | 0.0 | 8.9 | 10.2 | 95.9 | 44.9 | 1.2 | 5.1 | 1.2 | 0.0 | 0 |
| **50** | 21.0 | 7.7 | 0.0 | 8.3 | 9.9 | 90.5 | 57.3 | 3.7 | 3.6 | 1.4 | 0.0 | 0 |
| **51** | 20.7 | 2.0 | 0.0 | 6.0 | 8.7 | 93.2 | 49.6 | 1.4 | 6.3 | 1.2 | 0.0 | 0 |
| **52** | 19.8 | 1.9 | 0.0 | 5.9 | 8.0 | 94.1 | 48.6 | 1.8 | 4.4 | 0.9 | 0.0 | 0 |

2019

| **SMWs** | **T_max_**  **(°C)** | **T_min_**  **(°C)** | **G min**  **(°C)** | **AVP_m_**  **(kP)** | **AVP_e_**  **(kP)** | **RH_m_**  **(%)** | **RH_e_**  **(%)** | **WS**  **(km/hr)** | **BSS**  **(h)** | **PE**  **(mm)** | **RF**  **(mm)** | **RD**  **days** |
| --- | --- | --- | --- | --- | --- | --- | --- | --- | --- | --- | --- | --- |
| **1** | 18.9 | 5.7 | 2.0 | 8.0 | 9.8 | 95 | 66 | 3.4 | 3.3 | 0.9 | 7.3 | 1 |
| **2** | 19.3 | 5.6 | 1.5 | 8.0 | 9.8 | 93 | 60 | 2.4 | 4.4 | 0.9 | 0 | 0 |
| **3** | 20.4 | 4.9 | 1.6 | 7.2 | 9.7 | 90 | 55 | 2.3 | 5.0 | 1.0 | 0 | 0 |
| **4** | 18.2 | 4.8 | 2.6 | 7.5 | 9.2 | 99 | 63 | 3.1 | 4.6 | 1.1 | 6.5 | 1 |
| **5** | 17.1 | 5.3 | 3.8 | 7.4 | 9.4 | 96 | 65 | 3.6 | 3.9 | 1.1 | 0 | 0 |
| **6** | 21.0 | 6.9 | 4.4 | 8.3 | 10.1 | 92 | 56 | 3.9 | 5.7 | 1.6 | 0 | 0 |
| **7** | 20.0 | 9.7 | 7.9 | 9.8 | 11.4 | 94 | 67 | 4.0 | 3.1 | 1.2 | 0 | 0 |
| **8** | 22.2 | 9.0 | 5.6 | 9.0 | 10.0 | 89 | 50 | 5.1 | 5.6 | 2.1 | 0 | 0 |
| **9** | 20.9 | 8.0 | 0.0 | 8.8 | 9.3 | 93 | 53 | 5.0 | 5.8 | 1.9 | 14.8 | 2 |
| **10** | 24.2 | 8.5 | 0.0 | 9.2 | 9.1 | 88 | 38 | 3.8 | 8.2 | 2.7 | 0 | 0 |
| **11** | 24.9 | 9.1 | 0.0 | 10.5 | 10.9 | 91 | 48 | 3.7 | 6.1 | 2.6 | 0 | 0 |
| **12** | 28.9 | 11.8 | 0.0 | 10.4 | 12.4 | 80 | 42 | 4.2 | 7.2 | 4.0 | 0 | 0 |
| **13** | 32.6 | 13.5 | 0.0 | 14.4 | 10.6 | 81 | 28 | 3.2 | 7.3 | 3.7 | 0 | 0 |
| **14** | 36.0 | 16.0 | 0.0 | 15.3 | 12.7 | 74 | 28 | 3.8 | 8.0 | 5.4 | 7.3 | 1 |
| **15** | 36.9 | 20.0 | 0.0 | 16.1 | 12.6 | 69 | 27 | 5.6 | 7.0 | 6.4 | 0 | 0 |
| **16** | 32.9 | 17.2 | 0.0 | 15.4 | 12.3 | 81 | 37 | 5.2 | 7.2 | 5.8 | 8.2 | 2 |
| **17** | 40.7 | 20.6 | 0.0 | 15.3 | 10.6 | 56 | 19 | 5.3 | 8.5 | 8.3 | 0 | 0 |
| **18** | 40.1 | 20.8 | 0.0 | 12.7 | 12.4 | 48 | 23 | 6.0 | 9.3 | 8.9 | 0 | 0 |
| **19** | 40.5 | 21.7 | 0.0 | 13.4 | 10.9 | 48 | 20 | 5.5 | 7.9 | 8.0 | 0 | 0 |
| **20** | 35.7 | 20.5 | 0.0 | 17.8 | 14.8 | 82 | 37 | 6.0 | 8.3 | 6.5 | 59.8 | 5 |
| **21** | 38.0 | 22.7 | 0.0 | 16.7 | 13.0 | 61 | 26 | 4.7 | 8.9 | 6.2 | 0 | 0 |
| **22** | 43.8 | 25.2 | 0.0 | 17.9 | 11.8 | 53 | 18 | 4.6 | 9.9 | 8.9 | 0 | 0 |
| **23** | 43.5 | 25.8 | 0.0 | 18.2 | 10.9 | 55 | 17 | 5.6 | 8.8 | 9.6 | 0 | 0 |
| **24** | 41.8 | 26.0 | 0.0 | 18.8 | 15.9 | 67 | 36 | 7.4 | 8.3 | 8.6 | 55.1 | 3 |
| **25** | 35.7 | 24.6 | 0.0 | 21.9 | 18.9 | 78 | 43 | 6.7 | 8.2 | 5.9 | 20.3 | 1 |
| **26** | 39.1 | 25.5 | 0.0 | 23.7 | 22.3 | 77 | 44 | 5.5 | 7.6 | 6.1 | 29.7 | 1 |
| **27** | 38.6 | 26.2 | 0.0 | 23.9 | 23.5 | 74 | 56 | 6.5 | 6.5 | 6.7 | 1.8 | 0 |
| **28** | 37.2 | 27.3 | 0.0 | 23.2 | 23.3 | 73 | 57 | 9.9 | 0.7 | 6.1 | 13.4 | 1 |
| **29** | 32.5 | 23.7 | 0.0 | 24.4 | 25.7 | 93 | 70 | 6.2 | 4.5 | 3.5 | 81.5 | 2 |
| **30** | 33.2 | 25.3 | 0.0 | 25.8 | 25.9 | 88 | 73 | 6.4 | 3.8 | 3.4 | 23.7 | 2 |
| **31** | 33.8 | 25.1 | 0.0 | 26.7 | 25.6 | 87 | 67 | 5.6 | 5.3 | 4.7 | 17.7 | 2 |
| **32** | 34.6 | 27.0 | 0.0 | 25.7 | 25.4 | 87 | 68 | 7.2 | 6.3 | 4.7 | 63 | 1 |
| **33** | 33.3 | 25.5 | 0.0 | 24.8 | 25.0 | 89 | 70 | 5.7 | 4.5 | 3.4 | 15.4 | 1 |
| **34** | 35.6 | 25.8 | 0.0 | 24.1 | 23.5 | 86 | 56 | 4.7 | 8.2 | 4.4 | 0 | 0 |
| **35** | 36.2 | 27.4 | 0.0 | 26.7 | 23.6 | 84 | 55 | 5.2 | 6.9 | 5.0 | 0 | 0 |
| **36** | 35.7 | 27.0 | 0.0 | 27.1 | 25.1 | 89 | 59 | 4.1 | 6.2 | 4.1 | 27.7 | 1 |
| **37** | 37.0 | 27.1 | 0.0 | 26.3 | 23.1 | 88 | 51 | 5.1 | 6.6 | 5.0 | 0 | 0 |
| **38** | 35.1 | 25.2 | 0.0 | 23.1 | 20.0 | 84 | 49 | 6.8 | 6.7 | 5.1 | 0 | 0 |
| **39** | 33.4 | 24.3 | 0.0 | 21.7 | 20.6 | 84 | 58 | 6.9 | 7.2 | 4.6 | 2.2 | 0 |
| **40** | 31.8 | 21.0 | 0.0 | 19.6 | 17.1 | 92 | 50 | 4.1 | 5.5 | 3.7 | 2.6 | 1 |
| **41** | 32.7 | 17.6 | 0.0 | 16.7 | 14.0 | 90 | 39 | 2.9 | 7.5 | 3.9 | 0 | 0 |
| **42** | 34.1 | 18.4 | 0.0 | 15.8 | 12.8 | 79 | 34 | 4.5 | 7.3 | 4.0 | 0 | 0 |
| **43** | 31.9 | 14.9 | 0.0 | 13.7 | 10.8 | 79 | 31 | 2.1 | 6.5 | 3.1 | 0 | 0 |
| **44** | 30.7 | 16.2 | 0.0 | 14.2 | 12.1 | 90 | 40 | 1.3 | 1.8 | 1.8 | 0 | 0 |
| **45** | 28.4 | 12.7 | 0.0 | 11.2 | 10.0 | 85 | 36 | 4.1 | 6.6 | 2.8 | 0.3 | 0 |
| **46** | 26.8 | 12.7 | 0.0 | 11.8 | 10.5 | 86 | 41 | 3.1 | 2.5 | 2.0 | 0 | 0 |
| **47** | 26.7 | 10.9 | 0.0 | 10.5 | 10.5 | 88 | 42 | 2.5 | 4.7 | 2.3 | 0 | 0 |
| **48** | 22.6 | 12.1 | 0.0 | 11.2 | 11.8 | 92 | 62 | 3.5 | 2.8 | 1.5 | 12 | 2 |
| **49** | 23.1 | 6.0 | 2.2 | 7.3 | 9.5 | 88 | 47 | 1.5 | 6.2 | 1.3 | 0 | 0 |
| **50** | 19.2 | 8.3 | 5.4 | 9.0 | 10.2 | 95 | 74 | 3.8 | 2.2 | 1.2 | 4.5 | 1 |
| **51** | 13.7 | 6.1 | 3.9 | 8.1 | 9.4 | 99 | 81 | 3.2 | 1.1 | 0.9 | 0 | 0 |
| **52** | 11.9 | 2.6 | -0.7 | 6.7 | 7.4 | 97 | 75 | 3.1 | 1.7 | 0.8 | 0 | 0 |

2020

| **SMWs** | **T_max_**  **(°C)** | **T_min_**  **(°C)** | **G_min_**  **(°C)** | **AVP_m_**  **(kP)** | **AVP_e_**  **(kP)** | **RH_m_**  **(%)** | **RH_e_**  **(%)** | **WS**  **(km/hr)** | **BSS**  **(h)** | **PE**  **(mm)** | **RF**  **(mm)** |
| --- | --- | --- | --- | --- | --- | --- | --- | --- | --- | --- | --- |
| **1** | -1.5 | 1.1 | 0.5 | 0.6 | 0.9 | 3.0 | 5.8 | -0.4 | -1.9 | -0.5 | -2.4 |
| **2** | -1.4 | 1.3 | 0.6 | 0.6 | 0.6 | 3.8 | 12.3 | 0.0 | -2.7 | -0.5 | 0.4 |
| **3** | -5.8 | -0.1 | 0.7 | 0.3 | 1.2 | 8.0 | 30.4 | -0.9 | -4.0 | -1.3 | -2.8 |
| **4** | -0.9 | -0.2 | 0.2 | 0.3 | 0.9 | 4.9 | 6.1 | -0.2 | -0.8 | -0.6 | 3.9 |
| **5** | -2.1 | -1.5 | -0.9 | -0.5 | 0.9 | 6.1 | 12.8 | -0.4 | -0.7 | -0.8 | -2.0 |
| **6** | -1.7 | -3.0 | -3.1 | -1.6 | -0.9 | 2.6 | -1.8 | -2.0 | -0.4 | -0.7 | -4.6 |
| **7** | 1.9 | -2.8 | -3.9 | -1.6 | -1.0 | 3.0 | -11.7 | -1.0 | 1.5 | -0.3 | -4.2 |
| **8** | -0.2 | 2.5 | 3.1 | 1.0 | 3.0 | 1.0 | 15.5 | 1.5 | -1.3 | -0.5 | 6.0 |
| **9** | 0.8 | 3.3 | - | 2.4 | 3.4 | 6.9 | 12.0 | 1.1 | -1.4 | -1.1 | -2.3 |
| **10** | -4.1 | 1.8 | - | 1.2 | 2.2 | 4.1 | 25.9 | 1.0 | -2.2 | -1.1 | 57.6 |
| **11** | -4.5 | -1.4 | - | -0.3 | 0.9 | 8.3 | 13.0 | -1.0 | -1.6 | -1.5 | 7.8 |
| **12** | -0.9 | 1.9 | - | 1.7 | 4.6 | 7.8 | 16.2 | -1.0 | -2.0 | -1.2 | -1.2 |
| **13** | -4.1 | 1.2 | - | 3.0 | 4.0 | 12.6 | 22.5 | -0.6 | -3.4 | -1.9 | 18.6 |
| **14** | -2.6 | -1.6 | - | -0.9 | 2.3 | 7.1 | 9.6 | -1.9 | -0.9 | -2.2 | -3.0 |
| **15** | -0.4 | 1.3 | - | 1.6 | -0.2 | 6.0 | -2.4 | -2.4 | -1.8 | -2.6 | -3.8 |
| **16** | -0.9 | 0.6 | - | 1.8 | -1.0 | 9.2 | -1.7 | -1.0 | -1.1 | -2.3 | -2.8 |
| **17** | -4.3 | 0.2 | - | 2.3 | 2.9 | 16.6 | 17.7 | -0.5 | -1.7 | -4.0 | 2.6 |
| **18** | -1.9 | 1.1 | - | 3.6 | 2.4 | 14.9 | 8.5 | -0.2 | 0.2 | -2.8 | 13.9 |
| **19** | -2.2 | 0.5 | - | 1.7 | -0.2 | 8.8 | 3.2 | -1.0 | -0.3 | -3.2 | -4.2 |
| **20** | -1.8 | -2.4 | - | -0.4 | -2.6 | 3.6 | -4.9 | -3.9 | 0.7 | -3.8 | -1.6 |
| **21** | 2.5 | -0.7 | - | -0.7 | -2.2 | -5.2 | -8.6 | -3.5 | 2.1 | -0.3 | -5.8 |
| **22** | -4.0 | -1.5 | - | 2.2 | 4.8 | 17.9 | 20.1 | 0.5 | -0.2 | -3.8 | 2.5 |
| **23** | -4.4 | -0.8 | - | 3.4 | 3.4 | 15.9 | 12.4 | -3.6 | 0.3 | -5.1 | -4.7 |
| **24** | 1.5 | 1.4 | - | 3.0 | 1.1 | 3.7 | -4.1 | -4.7 | 2.3 | -2.5 | -17.4 |
| **25** | 0.5 | 0.5 | - | 2.5 | 1.5 | 7.9 | 1.0 | -2.2 | 1.1 | -2.5 | 12.9 |
| **26** | -1.0 | 1.4 | - | 2.8 | 3.1 | 2.5 | 6.0 | -1.4 | 0.6 | -2.6 | -9.6 |
| **27** | 1.0 | 1.2 | - | 2.5 | 1.4 | 4.4 | 3.2 | -0.9 | 2.3 | -0.8 | -15.5 |
| **28** | -0.3 | 0.1 | - | 2.7 | 3.4 | 9.1 | 5.9 | -2.3 | 2.3 | -0.6 | 12.2 |
| **29** | -1.0 | -0.2 | - | 1.3 | 3.1 | 8.9 | 15.9 | 0.2 | -0.4 | -1.1 | 69.4 |
| **30** | -0.7 | 0.5 | - | 2.2 | 1.6 | 3.7 | -1.8 | -3.7 | 1.2 | -1.8 | -17.7 |
| **31** | 1.6 | 0.9 | - | 2.0 | 3.0 | 0.7 | 3.8 | -2.1 | 0.7 | -1.2 | -31.5 |
| **32** | 0.5 | 1.6 | - | 2.4 | 2.4 | 3.0 | 0.4 | -0.2 | 0.3 | -0.6 | 6.4 |
| **33** | 0.5 | 1.3 | - | 3.1 | 4.9 | 5.0 | 15.8 | -0.3 | -0.2 | -1.6 | -2.7 |
| **34** | -2.5 | 0.0 | - | 1.6 | 2.5 | 6.2 | 16.2 | 1.3 | -1.3 | -1.1 | -13.7 |
| **35** | -1.1 | 0.7 | - | 1.9 | 1.3 | 6.9 | 6.2 | 1.0 | -1.1 | -1.1 | -16.0 |
| **36** | -0.6 | 1.0 | - | 4.0 | 4.3 | 10.5 | 11.6 | -3.2 | -2.9 | -2.3 | 15.0 |
| **37** | 1.6 | 1.5 | - | 2.8 | 1.9 | 4.0 | -4.1 | -2.4 | 0.3 | -0.9 | -22.0 |
| **38** | 2.9 | 2.3 | - | 3.1 | 2.5 | 3.4 | -1.8 | -1.1 | -0.8 | -0.5 | -4.4 |
| **39** | 2.4 | 1.6 | - | 2.0 | 0.8 | 2.6 | -5.4 | -0.7 | -1.2 | -0.4 | -9.6 |
| **40** | 2.0 | -1.2 | - | -0.9 | -2.1 | -2.8 | -10.6 | -0.8 | -0.6 | -0.4 | -3.5 |
| **41** | 1.2 | -1.4 | - | -0.8 | -1.4 | 4.6 | -6.7 | -0.8 | -0.9 | -0.5 | -1.0 |
| **42** | 0.9 | -2.2 | - | -1.4 | -2.7 | -4.9 | -10.4 | -1.0 | -1.5 | -0.8 | -3.3 |
| **43** | 0.9 | -1.2 | - | -1.2 | -1.1 | -2.3 | -4.7 | -0.2 | -1.8 | -0.1 | -0.2 |
| **44** | -0.8 | -3.0 | - | -2.1 | -2.6 | 1.8 | -8.2 | -0.4 | -2.0 | -0.7 | -1.5 |
| **45** | -0.3 | -1.0 | - | -0.5 | -0.4 | 6.4 | -1.5 | -1.7 | -4.3 | -0.9 | -0.4 |
| **46** | -2.9 | 2.0 | - | 1.3 | 1.2 | 2.6 | 12.9 | 0.9 | -4.6 | -1.3 | 18.2 |
| **47** | -4.0 | -1.1 | - | -0.3 | -0.7 | 0.4 | 6.3 | -0.7 | -1.1 | -1.3 | -0.2 |
| **48** | -2.2 | 1.2 | 3.4 | 0.3 | 0.8 | 3.0 | 4.7 | 0.2 | -0.8 | -0.2 | 0.2 |
| **49** | 1.0 | 2.8 | 1.5 | 2.1 | 4.0 | 1.5 | 14.9 | 0.1 | -1.5 | -0.6 | -0.5 |
| **50** | -1.9 | -1.0 | -0.8 | 1.0 | 1.3 | 4.5 | 18.2 | 0.3 | -1.2 | -0.5 | -1.1 |
| **51** | -1.6 | -2.3 | -2.9 | -0.8 | -0.8 | -0.1 | -5.7 | -0.7 | 0.0 | -0.4 | -1.0 |
| **52** | -0.3 | -1.9 | -2.1 | -0.7 | -0.3 | 2.2 | -2.4 | -0.4 | 0.2 | -0.2 | -2.3 |
