# Supplementary material for: The global invasion risk of rice yellow stem borer Scirpophaga incertulas Walker (Lepidoptera:Crambidae) under current and future climate scenarios
Source: PLoS One. 2025 Mar 5;20(3):e0310234. doi: 10.1371/journal.pone.0310234 (PMC11882091; doi:10.1371/journal.pone.0310234)
Supplement: S1 File — (ZIP) [file pone.0310234.s002.zip › weather data/Parbahni weather data.docx]

2018

| **WK** | **RF** | **Temperature ^o^C** | | **Humidity (%)** | | **EVP (mm)** | **BSS (Hrs.)** | **WS (Kmph)** |
| --- | --- | --- | --- | --- | --- | --- | --- | --- |
|  |  | **Max** | **Min** | **RH1** | **RH2** |  |  |  |
| 1 | 0.0 | 29.6 | 9.2 | 76 | 32 | 3.9 | 8.6 | 2.6 |
| 2 | 0.0 | 29.8 | 11.5 | 76 | 30 | 4.5 | 8.7 | 2.9 |
| 3 | 0.0 | 31.3 | 11.8 | 74 | 26 | 5.1 | 9.2 | 3.0 |
| 4 | 0.0 | 29.7 | 8.7 | 76 | 25 | 4.5 | 9.4 | 3.1 |
| 5 | 0.0 | 31.7 | 8.9 | 74 | 17 | 5.0 | 9.7 | 3.0 |
| 6 | 0.0 | 31.1 | 12.5 | 77 | 26 | 4.6 | 5.9 | 2.8 |
| 7 | 3.6 | 31.1 | 14.2 | 81 | 36 | 4.7 | 8.2 | 4.6 |
| 8 | 0.0 | 34.8 | 14.8 | 69 | 21 | 5.8 | 9.1 | 3.2 |
| 9 | 0.0 | 36.3 | 15.5 | 67 | 14 | 7.2 | 9.2 | 3.2 |
| 10 | 0.0 | 36.9 | 17.8 | 65 | 16 | 8.1 | 8.6 | 4.4 |
| 11 | 6.1 | 33.6 | 19.0 | 71 | 32 | 6.4 | 5.8 | 4.0 |
| 12 | 0.0 | 36.9 | 17.4 | 75 | 15 | 7.2 | 9.6 | 4.4 |
| 13 | 0.0 | 40.2 | 17.7 | 53 | 11 | 9.5 | 9.8 | 3.7 |
| 14 | 0.0 | 39.9 | 19.9 | 52 | 14 | 10.0 | 8.6 | 3.6 |
| 15 | 0.0 | 38.4 | 21.9 | 53 | 18 | 9.9 | 8.9 | 4.2 |
| 16 | 0.0 | 40.9 | 22.4 | 48 | 16 | 11.2 | 10 | 5.2 |
| 17 | 0.0 | 41.9 | 20.9 | 41 | 13 | 12.4 | 10.8 | 3.9 |
| 18 | 0.0 | 42.7 | 25.2 | 42 | 13 | 13.2 | 10.2 | 4.5 |
| 19 | 0.0 | 42.7 | 26.2 | 40 | 17 | 13.2 | 9.9 | 4.6 |
| 20 | 0.0 | 42.2 | 25.9 | 44 | 18 | 13.9 | 9.4 | 6.1 |
| 21 | 0.0 | 42.1 | 26.8 | 45 | 23 | 13.4 | 6.9 | 5.5 |
| 22 | 21.0 | 40.0 | 26.5 | 61 | 32 | 10.4 | 6.9 | 7.1 |
| 23 | 112.8 | 33.1 | 21.7 | 89 | 59 | 4.8 | 4.2 | 5.9 |
| 24 | 53.2 | 36.1 | 23.8 | 78 | 41 | 9.0 | 8.1 | 8.1 |
| 25 | 112.5 | 34.0 | 22.8 | 84 | 57 | 5.6 | 6.6 | 5.2 |
| 26 | 26.3 | 31.8 | 22.3 | 84 | 57 | 4.5 | 4.4 | 4.4 |
| 27 | 48.3 | 31.8 | 22.4 | 87 | 64 | 4.0 | 4.4 | 5.2 |
| 28 | 39.9 | 28.9 | 21.9 | 92 | 80 | 2.4 | 0.3 | 5.0 |
| 29 | 103.4 | 29.6 | 21.8 | 88 | 76 | 2.8 | 2.2 | 5.4 |
| 30 | 2.2 | 29.9 | 21.9 | 83 | 65 | 3.1 | 1.5 | 4.9 |
| 31 | 0.0 | 32.8 | 21.7 | 79 | 50 | 5.3 | 6.7 | 5.3 |
| 32 | 7.2 | 30.5 | 22.0 | 84 | 68 | 4.9 | 2.1 | 6.3 |
| 33 | 148.4 | 28.3 | 21.5 | 90 | 75 | 3.2 | 0.8 | 5.5 |
| 34 | 110.2 | 28.7 | 20.5 | 91 | 73 | 3.1 | 4.8 | 5.8 |
| 35 | 8.8 | 29.7 | 20.8 | 84 | 61 | 3.6 | 4.9 | 4.8 |
| 36 | 2.4 | 30.9 | 20.2 | 83 | 54 | 5.1 | 7.6 | 5.4 |
| 37 | 0.0 | 33.0 | 20.7 | 83 | 49 | 5.9 | 9.1 | 2.9 |
| 38 | 1.8 | 32.4 | 21.2 | 83 | 56 | 4.6 | 3.5 | 4.5 |
| 39 | 4.0 | 34.6 | 20.8 | 82 | 41 | 5.7 | 8.2 | 3.2 |
| 40 | 0.0 | 34.9 | 20.0 | 78 | 40 | 6.2 | 9.0 | 3.4 |
| 41 | 0.0 | 35.5 | 16.3 | 72 | 20 | 6.6 | 8.7 | 4.3 |
| 42 | 0.0 | 34.5 | 16.5 | 75 | 27 | 6.2 | 8.8 | 3.2 |
| 43 | 0.0 | 34.7 | 16.2 | 72 | 26 | 5.7 | 8.7 | 2.8 |
| 44 | 0.0 | 32.9 | 14.8 | 73 | 30 | 5.9 | 9.7 | 4.8 |
| 45 | 0.0 | 33.9 | 16.3 | 71 | 33 | 5.5 | 8.4 | 2.8 |
| 46 | 0.0 | 33.3 | 11.4 | 75 | 23 | 4.9 | 9.5 | 2.9 |
| 47 | 0.0 | 32.2 | 16.3 | 77 | 35 | 4.5 | 7.5 | 3.6 |
| 48 | 0.0 | 30.4 | 10.2 | 77 | 24 | 4.3 | 8.6 | 2.8 |
| 49 | 0.0 | 31.0 | 14.3 | 76 | 35 | 4.3 | 7.3 | 2.8 |
| 50 | 0.0 | 30.3 | 13.5 | 75 | 34 | 4.6 | 7.7 | 4.3 |
| 51 | 0.0 | 27.1 | 9.9 | 76 | 34 | 5.1 | 7.6 | 4.5 |
| 52 | 0.0 | 28.5 | 8.5 | 75 | 21 | 4.8 | 9.0 | 3.9 |

2019

| **WK** | **RF** | **Temperature ^o^C** | | **Humidity (%)** | | **EVP (mm)** | **BSS (Hrs.)** | **WS (Kmph)** |
| --- | --- | --- | --- | --- | --- | --- | --- | --- |
|  |  | **Max** | **Min** | **RH1** | **RH2** |  |  |  |
| 1 | 0.0 | 30.4 | 7.9 | 75 | 19 | 4.2 | 9.2 | 2.5 |
| 2 | 0.0 | 29.5 | 9.5 | 76 | 28 | 4.4 | 8.6 | 2.8 |
| 3 | 0.0 | 31.0 | 11.0 | 77 | 25 | 4.4 | 8.4 | 2.7 |
| 4 | 0.0 | 30.1 | 13.8 | 75 | 37 | 4.8 | 6.5 | 4.4 |
| 5 | 0.0 | 29.4 | 10.7 | 76 | 22 | 5.2 | 8.9 | 4.4 |
| 6 | 0.0 | 30.8 | 9.8 | 73 | 20 | 5.5 | 9.2 | 4.3 |
| 7 | 0.0 | 33.7 | 13.1 | 73 | 21 | 6.0 | 8.7 | 4.6 |
| 8 | 0.0 | 36.4 | 16.2 | 70 | 19 | 6.9 | 9.4 | 3.9 |
| 9 | 0.0 | 29.9 | 12.5 | 55 | 15 | 6.8 | 8.4 | 4.1 |
| 10 | 0.0 | 35.5 | 14.9 | 65 | 15 | 7.8 | 9.6 | 3.9 |
| 11 | 0.0 | 38.1 | 18.8 | 63 | 15 | 9.5 | 9.2 | 4.3 |
| 12 | 0.0 | 38.8 | 18.9 | 52 | 19 | 10.5 | 9.3 | 4.0 |
| 13 | 0.0 | 41.1 | 19.9 | 51 | 16 | 11.4 | 9.3 | 3.9 |
| 14 | 0.0 | 40.9 | 21.3 | 40 | 12 | 12.1 | 8.6 | 4.6 |
| 15 | 1.6 | 42.0 | 22.2 | 38 | 9 | 11.8 | 9.6 | 4.5 |
| 16 | 0.0 | 39.3 | 21.4 | 40 | 14 | 9.4 | 9.3 | 4.4 |
| 17 | 0.0 | 43.9 | 24.5 | 36 | 8 | 13.6 | 9.3 | 5.1 |
| 18 | 0.0 | 41.6 | 24.2 | 39 | 11 | 13.3 | 9.8 | 6.1 |
| 19 | 0.0 | 41.8 | 26.1 | 35 | 14 | 13.6 | 9.7 | 6.4 |
| 20 | 0.0 | 42.4 | 24.8 | 41 | 12 | 13.3 | 10.3 | 5.3 |
| 21 | 0.0 | 44.1 | 27.1 | 38 | 14 | 14.1 | 10.3 | 5.4 |
| 22 | 0.0 | 43.9 | 28.7 | 36 | 14 | 14.6 | 8.6 | 5.9 |
| 23 | 33.7 | 40.9 | 24.8 | 76 | 24 | 8.5 | 5.9 | 6.8 |
| 24 | 0.0 | 39.3 | 25.2 | 62 | 30 | 10.4 | 9.8 | 7.9 |
| 25 | 10.5 | 35.3 | 24.7 | 69 | 48 | 8.6 | 6.4 | 7.3 |
| 26 | 46.9 | 33.2 | 22.7 | 85 | 59 | 4.9 | 4.8 | 5.6 |
| 27 | 10.6 | 33.2 | 23.1 | 76 | 58 | 5.1 | 2.7 | 8.3 |
| 28 | 34.2 | 33.5 | 22.6 | 83 | 49 | 5.5 | 6.7 | 7.0 |
| 29 | 11.2 | 34.2 | 22.9 | 79 | 46 | 6.6 | 7.7 | 5.8 |
| 30 | 64.3 | 30.6 | 22.6 | 81 | 62 | 4.5 | 4.1 | 6.3 |
| 31 | 85.4 | 28.1 | 21.8 | 92 | 85 | 2.1 | 1.2 | 6.6 |
| 32 | 62.2 | 30.5 | 22.0 | 89 | 65 | 4.0 | 3.5 | 6.2 |
| 33 | 9.7 | 32.3 | 21.5 | 80 | 57 | 4.6 | 5.4 | 4.6 |
| 34 | 1.2 | 32.2 | 22.0 | 80 | 56 | 5.6 | 6.5 | 6.0 |
| 35 | 78.0 | 31.2 | 21.5 | 88 | 59 | 4.6 | 5.6 | 4.7 |
| 36 | 13.2 | 30.1 | 21.6 | 83 | 70 | 2.9 | 2.0 | 4.7 |
| 37 | 86.4 | 30.0 | 21.2 | 88 | 68 | 2.8 | 4.7 | 5.4 |
| 38 | 118.8 | 30.9 | 21.9 | 94 | 67 | 2.3 | 5.1 | 3.5 |
| 39 | 35.6 | 31.3 | 21.1 | 92 | 62 | 3.7 | 6.5 | 3.3 |
| 40 | 21.2 | 31.4 | 20.5 | 88 | 60 | 3.8 | 7.3 | 2.8 |
| 41 | 5.1 | 31.5 | 20.1 | 87 | 53 | 4.1 | 7.0 | 2.7 |
| 42 | 121.4 | 30.1 | 18.6 | 82 | 55 | 3.5 | 5.9 | 4.1 |
| 43 | 100.0 | 29.7 | 20.6 | 82 | 63 | 2.4 | 4.3 | 3.6 |
| 44 | 13.0 | 30.4 | 20.7 | 84 | 62 | 3.2 | 6.9 | 3.8 |
| 45 | 0.0 | 31.4 | 18.4 | 89 | 48 | 3.4 | 8.6 | 1.6 |
| 46 | 0.0 | 30.0 | 14.7 | 77 | 45 | 3.7 | 8.3 | 2.7 |
| 47 | 0.0 | 30.0 | 13.2 | 81 | 44 | 3.4 | 8.5 | 2.1 |
| 48 | 0.0 | 30.1 | 15.4 | 80 | 46 | 3.8 | 7.9 | 2.6 |
| 49 | 0.0 | 28.9 | 14.8 | 76 | 46 | 4.2 | 7.4 | 3.5 |
| 50 | 0.0 | 30.0 | 15.6 | 86 | 44 | 3.6 | 7.1 | 2.5 |
| 51 | 0.0 | 28.3 | 14.9 | 88 | 45 | 3.3 | 6.1 | 2.9 |
| 52 | 4.4 | 26.2 | 15.2 | 81 | 49 | 2.7 | 3.0 | 4.1 |

2020

| **WK** | **RF (mm)** | **RD** | **Temperature (^o^C)** | | **Humidity (%)** | | **EVP (mm)** | **BSS (Hrs.)** | **WS (Kmph)** |
| --- | --- | --- | --- | --- | --- | --- | --- | --- | --- |
|  |  |  | **Tmax (^o^C)** | **Tmin (^o^C)** | **RH-I (%)** | **RH-II (%)** |  |  |  |
| 1 | 3.4 | 0 | 27.0 | 15.0 | 83 | 52 | 2.7 | 5.7 | 4.1 |
| 2 | 0.0 | 0 | 28.0 | 12.9 | 78 | 43 | 3.3 | 6.9 | 3.4 |
| 3 | 0.0 | 0 | 29.0 | 13.9 | 78 | 42 | 3.2 | 7.8 | 3.4 |
| 4 | 0.0 | 0 | 31.2 | 13.8 | 81 | 33 | 4.1 | 8.9 | 3.1 |
| 5 | 1.3 | 0 | 28.9 | 13.7 | 77 | 40 | 4.3 | 8.1 | 5.2 |
| 6 | 0.0 | 0 | 28.6 | 16.5 | 75 | 50 | 3.5 | 5.6 | 5.1 |
| 7 | 0.0 | 0 | 31.5 | 13.1 | 78 | 31 | 5.0 | 8.8 | 3.6 |
| 8 | 0.0 | 0 | 33.6 | 14.3 | 78 | 24 | 7.0 | 9.5 | 3.7 |
| 9 | 0.0 | 0 | 33.0 | 14.3 | 74 | 27 | 7.1 | 9.2 | 3.7 |
| 10 | 9.0 | 1 | 33.1 | 16.3 | 76 | 31 | 7.5 | 8.8 | 4.0 |
| 11 | 2.8 | 1 | 34.5 | 18.0 | 68 | 28 | 7.4 | 9.1 | 4.4 |
| 12 | 8.8 | 1 | 35.7 | 17.1 | 67 | 21 | 7.2 | 9.2 | 3.4 |
| 13 | 36.8 | 3 | 37.0 | 20.0 | 77 | 24 | 6.8 | 9.1 | 5.5 |
| 14 | 0.0 | 1 | 38.1 | 20.9 | 69 | 25 | 8.4 | 9.8 | 3.9 |
| 15 | 0.0 | 0 | 39.3 | 20.4 | 60 | 17 | 8.9 | 9.7 | 3.6 |
| 16 | 0.0 | 0 | 40.6 | 22.9 | 54 | 18 | 9.4 | 9.8 | 4.1 |
| 17 | 0.0 | 0 | 40.1 | 22.0 | 55 | 20 | 9.7 | 10.4 | 4.6 |
| 18 | 0.0 | 0 | 41.8 | 23.2 | 48 | 16 | 11.1 | 10.3 | 3.9 |
| 19 | 2.0 | 0 | 40.3 | 25.3 | 47 | 20 | 12.4 | 9.8 | 5.2 |
| 20 | 11.0 | 0 | 41.1 | 24.4 | 65 | 22 | 9.1 | 7.2 | 5.2 |
| 21 | 0.0 | 0 | 43.7 | 24.8 | 44 | 15 | 13.3 | 10.6 | 5.1 |
| 22 | 18.6 | 2 | 37.5 | 25.4 | 62 | 36 | 10.8 | 8.8 | 6.7 |
| 23 | 0.0 | 0 | 36.3 | 23.8 | 67 | 35 | 10.0 | 8.7 | 7.9 |
| 24 | 148.0 | 4 | 31.7 | 23.1 | 89 | 65 | 2.3 | 3.2 | 4.5 |
| 25 | 9.2 | 1 | 33.5 | 24.0 | 83 | 59 | 4.1 | 4.2 | 5.6 |
| 26 | 40.9 | 4 | 34.0 | 23.6 | 89 | 59 | 5.3 | 7.2 | 4.0 |
| 27 | 47.6 | 2 | 32.6 | 23.3 | 83 | 66 | 4.1 | 4.6 | 4.8 |
| 28 | 115.6 | 3 | 31.6 | 23.2 | 84 | 67 | 3.4 | 6.0 | 4.0 |
| 29 | 17.4 | 2 | 32.2 | 23.0 | 84 | 65 | 4.1 | 5.5 | 3.3 |
| 30 | 57.1 | 4 | 30.8 | 22.7 | 85 | 67 | 3.9 | 6.3 | 3.2 |
| 31 | 9.1 | 1 | 32.3 | 23.0 | 80 | 66 | 4.0 | 5.9 | 2.8 |
| 32 | 29.2 | 3 | 30.0 | 22.6 | 87 | 72 | 3.5 | 3.6 | 4.2 |
| 33 | 42.5 | 4 | 27.9 | 22.0 | 95 | 79 | 1.4 | 0.3 | 4.0 |
| 34 | 28.8 | 4 | 30.6 | 21.6 | 92 | 68 | 2.6 | 5.0 | 3.4 |
| 35 | 24.2 | 1 | 31.0 | 21.8 | 90 | 65 | 3.3 | 5.7 | 3.5 |
| 36 | 19.8 | 1 | 33.8 | 21.9 | 87 | 53 | 4.9 | 8.2 | 2.7 |
| 37 | 63.6 | 5 | 31.4 | 22.4 | 91 | 68 | 3.2 | 4.5 | 3.2 |
| 38 | 207.2 | 6 | 31.2 | 22.3 | 95 | 75 | 2.0 | 2.7 | 3.1 |
| 39 | 27.8 | 2 | 30.5 | 21.9 | 89 | 66 | 3.8 | 4.9 | 3.4 |
| 40 | 17.0 | 1 | 33.2 | 21.2 | 88 | 45 | 4.9 | 7.7 | 3.9 |
| 41 | 89.2 | 4 | 31.1 | 21.5 | 91 | 69 | 2.8 | 3.3 | 2.8 |
| 42 | 4.4 | 0 | 31.9 | 22.2 | 91 | 56 | 3.9 | 6.8 | 3.9 |
| 43 | 6.4 | 1 | 32.2 | 19.9 | 90 | 44 | 3.8 | 6.4 | 2.3 |
| 44 | 0.0 | 0 | 32.4 | 15.1 | 87 | 28 | 4.9 | 9.0 | 2.1 |
| 45 | 0.0 | 0 | 30.6 | 10.3 | 84 | 23 | 5.2 | 9.3 | 2.9 |
| 46 | 0.0 | 0 | 32.0 | 14.8 | 84 | 35 | 4.8 | 9.3 | 3.1 |
| 47 | 0.0 | 0 | 31.6 | 16.2 | 83 | 42 | 4.1 | 8.0 | 2.6 |
| 48 | 0.0 | 0 | 29.9 | 15.5 | 81 | 43 | 4.6 | 6.7 | 4.7 |
| 49 | 0.0 | 0 | 31.1 | 9.0 | 86 | 25 | 4.9 | 9.4 | 2.8 |
| 50 | 0.0 | 0 | 30.2 | 15.3 | 83 | 38 | 4.0 | 6.9 | 2.8 |
| 51 | 0.0 | 0 | 28.5 | 9.0 | 89 | 33 | 3.9 | 8.2 | 2.9 |
| 52 | 0.0 | 0 | 29.0 | 10.3 | 88 | 34 | 4.1 | 8.0 | 2.5 |

2021

| **WK** | **RF (mm)** | **RD** | **Temperature (^o^C)** | | **Humidity (%)** | | **EVP (mm)** | **BSS (Hrs.)** | **WS (Kmph)** |
| --- | --- | --- | --- | --- | --- | --- | --- | --- | --- |
|  |  |  | **Tmax (^o^C)** | **Tmin (^o^C)** | **RH-I (%)** | **RH-II (%)** |  |  |  |
| 1 | 0.0 | 0.0 | 28.8 | 15.4 | 88 | 51 | 3.7 | 4.3 | 3.8 |
| 2 | 0.0 | 0.0 | 31.0 | 15.3 | 90 | 40 | 4.4 | 6.3 | 3.5 |
| 3 | 0.0 | 0.0 | 31.2 | 15.1 | 82 | 35 | 4.4 | 7.6 | 3.1 |
| 4 | 0.0 | 0.0 | 31.7 | 13.6 | 83 | 34 | 4.3 | 8.2 | 2.5 |
| 5 | 0.0 | 0.0 | 30.3 | 12.9 | 78 | 29 | 5.2 | 7.4 | 3.4 |
| 6 | 0.0 | 0.0 | 30.2 | 11.3 | 66 | 20 | 5.9 | 9.4 | 3.0 |
| 7 | 1.8 | 0.0 | 32.4 | 14.3 | 76 | 24 | 5.6 | 8.7 | 2.8 |
| 8 | 14.5 | 2.0 | 30.3 | 13.0 | 93 | 40 | 4.5 | 7.7 | 3.4 |
| 9 | 0.0 | 0.0 | 36.3 | 15.5 | 67 | 14 | 7.2 | 9.1 | 2.9 |
| 10 | 0.0 | 0.0 | 36.6 | 16.6 | 61 | 14 | 7.1 | 9.3 | 2.6 |
| 11 | 0.0 | 0.0 | 36.8 | 16.8 | 60 | 21 | 7.5 | 9.0 | 3.0 |
| 12 | 14.3 | 2.0 | 34.9 | 20.1 | 74 | 28 | 6.9 | 7.0 | 5.0 |
| 13 | 0.0 | 0.0 | 39.3 | 16.0 | 57 | 12 | 9.1 | 9.2 | 3.3 |
| 14 | 0.0 | 0.0 | 39.4 | 19.2 | 48 | 11 | 10.1 | 9.2 | 3.8 |
| 15 | 2.0 | 0.0 | 36.3 | 20.5 | 63 | 25 | 7.0 | 5.5 | 4.4 |
| 16 | 0.0 | 0.0 | 39.5 | 19.9 | 51 | 13 | 9.6 | 9.2 | 3.5 |
| 17 | 0.0 | 0.0 | 39.8 | 21.2 | 46 | 13 | 9.7 | 9.1 | 4.1 |
| 18 | 8.2 | 1.0 | 38.8 | 22.8 | 61 | 20 | 8.7 | 8.7 | 4.2 |
| 19 | 0.0 | 0.0 | 38.8 | 24.2 | 59 | 26 | 8.9 | 8.3 | 4.1 |
| 20 | 3.0 | 0.0 | 37.9 | 25.7 | 60 | 31 | 9.4 | 7.6 | 7.8 |
| 21 | 0.0 | 0.0 | 39.8 | 25.3 | 50 | 20 | 11.9 | 8.4 | 7.2 |
| 22 | 71.1 | 4.0 | 35.7 | 23.0 | 76 | 43 | 7.6 | 6.1 | 5.5 |
| 23 | 154.4 | 5.0 | 32.9 | 21.0 | 87 | 60 | 3.3 | 5.7 | 3.5 |
| 24 | 102.6 | 4.0 | 32.5 | 21.5 | 88 | 62 | 4.6 | 6.0 | 5.8 |
| 25 | 9.7 | 2.0 | 32.7 | 19.6 | 86 | 57 | 4.7 | 5.5 | 5.4 |
| 26 | 35.3 | 1.0 | 32.9 | 23.3 | 84 | 57 | 4.6 | 5.6 | 5.2 |
| 27 | 41.1 | 2.0 | 33.4 | 23.8 | 82 | 54 | 5.3 | 5.9 | 4.2 |
| 28 | 389.7 | 6.0 | 29.7 | 22.0 | 96 | 78 | 1.1 | 2.4 | 3.7 |
| 29 | 126.7 | 4.0 | 30.1 | 22.6 | 92 | 73 | 3.0 | 5.7 | 4.1 |
| 30 | 9.9 | 2.0 | 30.5 | 21.4 | 89 | 65 | 3.4 | 4.5 | 5.3 |
| 31 | 2.4 | 0.0 | 30.9 | 21.6 | 84 | 63 | 3.3 | 2.7 | 5.8 |
| 32 | 2.3 | 0.0 | 33.1 | 22.5 | 84 | 52 | 4.9 | 6.2 | 4.2 |
| 33 | 48.5 | 4.0 | 29.4 | 22.2 | 89 | 70 | 3.6 | 4.7 | 4.6 |
| 34 | 5.9 | 1.0 | 30.6 | 22.4 | 92 | 64 | 3.1 | 5.2 | 2.9 |
| 35 | 48.8 | 3.0 | 30.0 | 22.7 | 91 | 68 | 3.0 | 3.4 | 3.1 |
| 36 | 233.1 | 5.0 | 28.2 | 21.8 | 92 | 79 | 1.6 | 3.9 | 3.7 |
| 37 | 44.4 | 3.0 | 30.9 | 22.0 | 91 | 67 | 3.4 | 6.6 | 4.1 |
| 38 | 48.6 | 3.0 | 30.9 | 22.3 | 92 | 64 | 4.0 | 5.1 | 3.6 |
| 39 | 133.9 | 5.0 | 28.9 | 21.8 | 94 | 75 | 1.6 | 2.2 | 3.6 |
| 40 | 112.9 | 3.0 | 32.7 | 22.4 | 94 | 59 | 3.5 | 7.3 | 2.4 |
| 41 | 3.0 | 0.0 | 33.0 | 21.2 | 92 | 46 | 4.4 | 7.8 | 2.3 |
| 42 | 45.8 | 1.0 | 31.1 | 19.6 | 89 | 48 | 4.2 | 7.0 | 2.9 |
| 43 | 0.0 | 0.0 | 31.5 | 15.9 | 86 | 30 | 5.0 | 9.4 | 2.1 |
| 44 | 0.0 | 0.0 | 31.2 | 15.7 | 79 | 36 | 5.5 | 8.5 | 3.7 |
| 45 | 0.0 | 0.0 | 30.9 | 14.3 | 85 | 29 | 5.0 | 7.6 | 3.3 |
| 46 | 0.0 | 0.0 | 30.8 | 20.6 | 81 | 54 | 4.1 | 4.5 | 4.6 |
| 47 | 1.2 | 0.0 | 31.7 | 21.7 | 88 | 49 | 4.0 | 6.5 | 4.3 |
| 48 | 0.0 | 0.0 | 28.8 | 15.3 | 79 | 35 | 5.0 | 5.9 | 3.9 |
| 49 | 4.2 | 1.0 | 28.2 | 16.7 | 87 | 45 | 3.1 | 3.5 | 3.0 |
| 50 | 0.0 | 0.0 | 28.7 | 13.2 | 88 | 35 | 4.7 | 5.9 | 3.0 |
| 51 | 0.0 | 0.0 | 28.1 | 9.4 | 91 | 30 | 3.4 | 7.1 | 2.3 |
| 52 | 0.0 | 0.0 | 28.2 | 13.6 | 88 | 44 | 3.1 | 4.9 | 3.0 |
| Mean/Total | **1719.3** | **64.0** | **32.7** | **19.1** | **79** | **42** | **5.2** | **6.6** | **3.9** |

2022
