# Supplementary material for: The global invasion risk of rice yellow stem borer Scirpophaga incertulas Walker (Lepidoptera:Crambidae) under current and future climate scenarios
Source: PLoS One. 2025 Mar 5;20(3):e0310234. doi: 10.1371/journal.pone.0310234 (PMC11882091; doi:10.1371/journal.pone.0310234)
Supplement: S1 File — (ZIP) [file pone.0310234.s002.zip › weather data/Raipur Weather doc (2).docx]

2018

kharif

| **Weeks No.** | **Dates** | Tmax-2018 | Tmax-Normal | Tmin-  2018 | Tmin-Normal | Rainfall-2018 | Rainfall-Normal |
| --- | --- | --- | --- | --- | --- | --- | --- |
| **21** | **May 21-27** | 41.7 | 42.5 | 28.9 | 27.4 | 0 | 5.3 |
| **22** | **May 28- 3 Jun** | 40.5 | 42.3 | 28 | 27.6 | 3 | 6.6 |
| **23** | **Jun 04-10** | 38.3 | 40.6 | 26.7 | 27.5 | 13.4 | 17.5 |
| **24** | **11-17** | 37.3 | 38.0 | 26.2 | 26.2 | 81.8 | 47.5 |
| **25** | **18-24** | 38 | 34.8 | 26.6 | 25.2 | 26.4 | 59.3 |
| **26** | **25-01** | 33.5 | 32.8 | 26 | 24.6 | 16.9 | 71.4 |
| **27** | **Jul 02-08** | 33.8 | 32.3 | 25.4 | 24.4 | 1.6 | 64.9 |
| **28** | **09-15** | 31.1 | 31.2 | 25 | 24.1 | 199.2 | 84.8 |
| **29** | **16-22** | 30.4 | 30.5 | 25.4 | 24.1 | 75.8 | 82.8 |
| **30** | **23-29** | 28.6 | 30.4 | 25 | 24.1 | 51.4 | 71.7 |
| **31** | **30-05** | 31.9 | 30.3 | 25.4 | 24.2 | 31 | 86.2 |
| **32** | **Aug 06-12** | 30 | 29.8 | 24.8 | 24.1 | 103.4 | 77.8 |
| **33** | **13-19** | 30.3 | 29.9 | 25.3 | 24.1 | 101.2 | 89.2 |
| **34** | **20-26** | 29 | 30.1 | 24.6 | 24.1 | 60.4 | 53.6 |
| **35** | **27-02** | 28.3 | 30.1 | 24.1 | 24.1 | 275 | 69.5 |
| **36** | **Sep 03-09** | 29.2 | 30.4 | 23.9 | 24 | 30.2 | 57.2 |
| **37** | **10-16** | 32.6 | 30.9 | 25.1 | 24 | 0 | 55.5 |
| **38** | **17-23** | 31 | 31.4 | 24.1 | 24.1 | 32.8 | 38.6 |
| **39** | **24-30** | 32.9 | 31.7 | 25 | 23.8 | 11 | 24.2 |
| **40** | **Oct 01-07** | 34 | 31.5 | 23.8 | 23.1 | 0 | 16.4 |
| **41** | **08-14** | 32.4 | 31.7 | 22.8 | 22.2 | 0 | 10.8 |
| **42** | **15-21** | 33.4 | 31.2 | 21.3 | 20.6 | 0 | 9 |
| **43** | **22-28** | 32.9 | 30.5 | 18.9 | 18.8 | 0 | 10.4 |
| **44** | **29-04** | 31 | 29.8 | 19.6 | 17.7 | 0 | 4 |
| **45** | **Nov 05-11** | 32.3 | 29.8 | 17.7 | 16.3 | 0 | 3.9 |
| **46** | **12-18** | 31.5 | 29.6 | 14.4 | 15.2 | 0 | 1.9 |
| **47** | **19-25** | 31.4 | 29.1 | 15.3 | 14 | 0 | 3 |
| **48** | **26-02** | 29.3 | 28.5 | 13.6 | 12.8 | 0 | 2.4 |
|  | **Mean / Total** | **32.7** | 32.2 | **23.3** | 22.6 | **1114.5** | 1125.4 |

Rabi

| **Week No.** | **Max. Temp. (2018-19)** | **Min. Temp. (2018-19)** | **Rainfall (2018-19)** | **Max. Temp. (Normal)** | **Min. Temp. (Normal)** | **Rainfall (Normal)** |
| --- | --- | --- | --- | --- | --- | --- |
|  |  |  |  |  |  |  |
| **46** | 31.5 | 14.4 | 0 | 29.5 | 15.1 | 2.6 |
| **47** | 31.4 | 15.3 | 0 | 29 | 13.9 | 2.6 |
| **48** | 29.3 | 13.6 | 0 | 28.3 | 12.6 | 2.9 |
| **49** | 28.2 | 14.3 | 0 | 27.8 | 11.5 | 0.5 |
| **50** | 27.4 | 15.7 | 0 | 27.5 | 11.2 | 1.5 |
| **51** | 22.1 | 11 | 47.2 | 27.3 | 10.6 | 0.8 |
| **52** | 25.2 | 8.5 | 0 | 27.1 | 10.8 | 1.5 |
| **1** | 27.4 | 8.5 | 0 | 26.6 | 10.1 | 2.2 |
| **2** | 27.1 | 10.2 | 0 | 27.2 | 11.3 | 3.8 |
| **3** | 28.1 | 9.2 | 0 | 27.7 | 11.5 | 2 |
| **4** | 26.3 | 14.3 | 23.6 | 28.1 | 11.7 | 3.8 |
| **5** | 26.4 | 9.5 | 0 | 28.8 | 12.7 | 4.3 |
| **6** | 28.8 | 12.5 | 3.4 | 29.1 | 12.9 | 4.4 |
| **7** | 30.2 | 13.6 | 9 | 30.3 | 14.1 | 4.2 |
| **8** | 33.1 | 17 | 0 | 31.5 | 14.5 | 1.5 |
| **9** | 31.0 | 17.3 | 0.2 | 32.6 | 15.3 | 4.1 |
| **10** | 33.3 | 17.6 | 0 | 33.7 | 16.6 | 3.8 |
| **11** | 35.6 | 21.6 | 0 | 34.8 | 17.5 | 1.7 |
| **12** | 34.5 | 19.8 | 9.2 | 36.8 | 18.7 | 2 |
| **13** | 38.2 | 20.6 | 10.8 | 37.3 | 19.6 | 2.5 |
| **Mean/**  **Total** | **29.8** | **14.2** | **103.4** | **30.1** | **13.6** | **52.7** |

2019

Kharif

| **Wk No.** | **Date** | **Tmax.**  **2019** | **Tmax.**  **Normal** | **Tmin.**  **2019** | **Tmax.**  **Normal** | **Rainfall**  **2019** | **Rainfall-Normal** |
| --- | --- | --- | --- | --- | --- | --- | --- |
| **21** | **21-27** | 44.2 | 42.5 | 29.5 | 27.4 | 0.0 | 5.3 |
| **22** | **28-03** | 43.4 | 42.3 | 29.4 | 27.6 | 3.2 | 6.6 |
| **23** | **Jun 04-10** | 42.7 | 40.6 | 28.6 | 27.5 | 5.2 | 17.5 |
| **24** | **11-17** | 43.5 | 38.0 | 29.7 | 26.2 | 22.0 | 47.5 |
| **25** | **18-24** | 37.4 | 34.8 | 26.3 | 25.2 | 45.4 | 59.3 |
| **26** | **25-01** | 36.8 | 32.8 | 27.5 | 24.6 | 2.0 | 71.4 |
| **27** | **Jul 02-08** | 29.5 | 32.3 | 24.7 | 24.4 | 69.6 | 64.9 |
| **28** | **09-15** | 33.5 | 31.2 | 26.1 | 24.1 | 13.2 | 84.8 |
| **29** | **16-22** | 35.5 | 30.5 | 26.4 | 24.1 | 26.1 | 82.8 |
| **30** | **23-29** | 32.3 | 30.4 | 25.5 | 24.1 | 8.4 | 71.7 |
| **31** | **30-05** | 28.0 | 30.3 | 24.4 | 24.2 | 99.0 | 86.2 |
| **32** | **Aug 06-12** | 30.2 | 29.8 | 25.3 | 24.1 | 185.6 | 77.8 |
| **33** | **13-19** | 30.8 | 29.9 | 25.4 | 24.1 | 49.2 | 89.2 |
| **34** | **20-26** | 31.7 | 30.1 | 25.2 | 24.1 | 27.8 | 53.6 |
| **35** | **27-02** | 30.9 | 30.1 | 25.1 | 24.1 | 53.8 | 69.5 |
| **36** | **Sep 03-09** | 29.5 | 30.4 | 24.9 | 24 | 239.0 | 57.2 |
| **37** | **10-16** | 30.7 | 30.9 | 25.0 | 24 | 9.2 | 55.5 |
| **38** | **17-23** | 33.0 | 31.4 | 25.5 | 24.1 | 2.4 | 38.6 |
| **39** | **24-30** | 30.2 | 31.7 | 24.2 | 23.8 | 178.1 | 24.2 |
| **40** | **Oct 01-07** | 32.0 | 31.5 | 24.3 | 23.1 | 1.8 | 16.4 |
| **41** | **08-14** | 31.3 | 31.7 | 23.6 | 22.2 | 1.2 | 10.8 |
| **42** | **15-21** | 30.9 | 31.2 | 21.8 | 20.6 | 51.0 | 9.0 |
| **43** | **22-28** | 28.1 | 30.5 | 22.2 | 18.8 | 27.6 | 10.4 |
| **44** | **29-04** | 31.4 | 29.8 | 22.2 | 17.7 | 0.0 | 4.0 |
| **45** | **Nov 05-11** | 30.6 | 29.8 | 23.0 | 16.3 | 81.6 | 3.9 |
| **46** | **12-18** | 29.6 | 29.6 | 15.5 | 15.2 | 0.0 | 1.9 |
| **47** | **19-25** | 30.2 | 29.1 | 15.2 | 14 | 0.0 | 3.0 |
| **48** | **26-02** | 29.7 | 28.5 | 16.3 | 12.8 | 0.0 | 2.4 |
|  | **Mean / Total** | **33.1** | **32.2** | **24.4** | **22.6** | **1202.4** | **1125.4** |

Rabi

| **Week No.** | **Max. Temp. (2019-20)** | **Min. Temp. (2019-20)** | **Rainfall (2019-20)** | **Max. Temp. (Normal)** | **Min. Temp. (Normal)** | **Rainfall (Normal)** |
| --- | --- | --- | --- | --- | --- | --- |
|  |  |  |  |  |  |  |
| **46** | 29.6 | 15.5 | 0 | 29.5 | 15.1 | 2.6 |
| **47** | 30.2 | 15.2 | 0 | 29.0 | 13.9 | 2.6 |
| **48** | 29.7 | 16.3 | 0 | 28.3 | 12.6 | 2.9 |
| **49** | 28 | 13.3 | 0 | 27.8 | 11.5 | 0.5 |
| **50** | 29.5 | 15.3 | 0 | 27.5 | 11.2 | 1.5 |
| **51** | 26.7 | 14.1 | 0.8 | 27.3 | 10.6 | 0.8 |
| **52** | 26.1 | 11.9 | 0 | 27.1 | 10.8 | 1.5 |
| **1** | 23.3 | 12.9 | 19.4 | 26.6 | 10.1 | 2.2 |
| **2** | 25.1 | 10.8 | 3.2 | 27.2 | 11.3 | 3.8 |
| **3** | 28.6 | 14.1 | 0 | 27.7 | 11.5 | 2 |
| **4** | 28.8 | 13.4 | 0 | 28.1 | 11.7 | 3.8 |
| **5** | 26.1 | 13.9 | 0 | 28.8 | 12.7 | 4.3 |
| **6** | 21.3 | 13.6 | 49.6 | 29.1 | 12.9 | 4.4 |
| **7** | 29.7 | 11.9 | 0 | 30.3 | 14.1 | 4.2 |
| **8** | 31.4 | 15.5 | 35.8 | 31.5 | 14.5 | 1.5 |
| **9** | 30 | 16.3 | 0.2 | 32.6 | 15.3 | 4.1 |
| **10** | 30.1 | 19.4 | 1.8 | 33.7 | 16.6 | 3.8 |
| **11** | 31.2 | 20.8 | 37.2 | 34.8 | 17.5 | 1.7 |
| **12** | 33.6 | 20 | 1.6 | 36.8 | 18.7 | 2 |
| **13** | 35.1 | 21.9 | 8.4 | 37.3 | 19.6 | 2.5 |
| **14** | 37.5 | 22 | 1 | 38.3 | 21.1 | 4.1 |
| **Mean/**  **Total** | **29.1** | **15.6** | **159** | **30.4** | **14.0** | **56.8** |

2020

Kharif

| **Wk No.** | **Date** | **Tmax.**  **2020** | **Tmax.**  **Normal** | **Tmin.**  **2020** | **Tmin..**  **Normal** | **Rainfall**  **2020** | **Rainfall-Normal** |
| --- | --- | --- | --- | --- | --- | --- | --- |
| **21** | **May 21-27** | 43.8 | 42.5 | 25.7 | 27.4 | 0.0 | 5.3 |
| **22** | **May 28-03 Jun** | 40.6 | 42.3 | 25.8 | 27.6 | 30.4 | 6.6 |
| **23** | **Jun 04-10** | 37.1 | 40.6 | 25.5 | 27.5 | 44.2 | 17.5 |
| **24** | **11-17** | 34.3 | 38.0 | 25.9 | 26.2 | 62.0 | 47.5 |
| **25** | **18-24** | 30.9 | 34.8 | 24.5 | 25.2 | 156.8 | 59.3 |
| **26** | **25-01** | 33.9 | 32.8 | 26.0 | 24.6 | 20.6 | 71.4 |
| **27** | **Jul 02-08** | 33.6 | 32.3 | 25.8 | 24.4 | 95.0 | 64.9 |
| **28** | **09-15** | 33.4 | 31.2 | 25.0 | 24.1 | 67.4 | 84.8 |
| **29** | **16-22** | 33.0 | 30.5 | 26.1 | 24.1 | 39.8 | 82.8 |
| **30** | **23-29** | 32.3 | 30.4 | 25.9 | 24.1 | 29.9 | 71.7 |
| **31** | **30-05 Aug** | 33.3 | 30.3 | 26.0 | 24.2 | 23.6 | 86.2 |
| **32** | **Aug 06-12** | 30.5 | 29.8 | 25.5 | 24.1 | 81.6 | 77.8 |
| **33** | **13-19** | 28.4 | 29.9 | 25.1 | 24.1 | 71.2 | 89.2 |
| **34** | **20-26** | 31.0 | 30.1 | 25.5 | 24.1 | 29.8 | 53.6 |
| **35** | **27-02 Sep** | 29.8 | 30.1 | 24.6 | 24.1 | 235.8 | 69.5 |
| **36** | **Sep 03-09** | 32.9 | 30.4 | 26.0 | 24 | 8.0 | 57.2 |
| **37** | **10-16** | 33.2 | 30.9 | 26.0 | 24 | 64.0 | 55.5 |
| **38** | **17-23** | 32.7 | 31.4 | 25.8 | 24.1 | 16.4 | 38.6 |
| **39** | **24-30** | 32.8 | 31.7 | 25.3 | 23.8 | 0.0 | 24.2 |
| **40** | **Oct 01-07** | 31.8 | 31.5 | 25.0 | 23.1 | 12.8 | 16.4 |
| **41** | **08-14** | 32.5 | 31.7 | 25.3 | 22.2 | 0.0 | 10.8 |
| **42** | **15-21** | 31.9 | 31.2 | 24.4 | 20.6 | 7.0 | 9.0 |
| **43** | **22-28** | 32.6 | 30.5 | 20.1 | 18.8 | 0.0 | 10.4 |
| **44** | **29-04 Nov** | 31.8 | 29.8 | 17.4 | 17.7 | 0 | 4.0 |
| **45** | **Nov 05-11** | 30.3 | 29.8 | 12.5 | 16.3 | 0 | 3.9 |
| **46** | **12-18** | 32.7 | 29.6 | 18.9 | 15.2 | 0 | 1.9 |
| **47** | **19-25** | 31 | 29.1 | 15.9 | 14.0 | 0 | 3.0 |
| **48** | **26-02 Dec** | 28.6 | 28.5 | 14.7 | 12.8 | 0 | 2.4 |
|  | **Mean / Total** | **32.9** | **32.2** | **23.6** | **22.6** | **1096.3** | **1125.4** |

Rabi

| **Week No.** | **Dates** | **Max. Temp. (2020-21)** | **Min. Temp. (2020-21)** | **Rainfall**  **(2020-21)** | **Max. Temp. (Normal)** | **Min. Temp. (Normal)** | **Rainfall (Normal)** |
| --- | --- | --- | --- | --- | --- | --- | --- |
|  |  |  |  |  |  |  |  |
| **46** | 3-9 Dec | 32.7 | 18.9 | 0.0 | 29.5 | 15.1 | 2.6 |
| **47** | 10-16 Dec | 31.0 | 15.9 | 0.4 | 29.0 | 13.9 | 2.6 |
| **48** | 17-23 Dec | 28.6 | 14.7 | 0.0 | 28.3 | 12.6 | 2.9 |
| **49** | 24-31 Dec | 30.7 | 11.9 | 0.0 | 27.8 | 11.5 | 0.5 |
| **50** | 1-7 Jan | 30.2 | 15.8 | 0.0 | 27.5 | 11.2 | 1.5 |
| **51** | 8-14 Jan | 27.7 | 10.3 | 0.0 | 27.3 | 10.6 | 0.8 |
| **52** | 15-21 Jan | 28.4 | 10.3 | 0.0 | 27.1 | 10.8 | 1.5 |
| **1** | 22-28 Jan | 29.7 | 13.8 | 0.0 | 26.6 | 10.1 | 2.2 |
| **2** | 29 Jan-4 Feb | 31.2 | 16.3 | 0.0 | 27.2 | 11.3 | 3.8 |
| **3** | 5-11 Feb | 30.0 | 11.7 | 0.0 | 27.7 | 11.5 | 2 |
| **4** | 12-18 Feb | 31.0 | 14.2 | 0.0 | 28.1 | 11.7 | 3.8 |
| **5** | 19-25 Feb | 28.2 | 10.3 | 4.6 | 28.8 | 12.7 | 4.3 |
| **6** | 26 Feb-4 Mar | 30.1 | 10.6 | 0.0 | 29.1 | 12.9 | 4.4 |
| **7** | 5 -11 Mar | 31.4 | 14.9 | 1.0 | 30.3 | 14.1 | 4.2 |
| **8** | 12-18 Mar | 30.9 | 14.5 | 1.4 | 31.5 | 14.5 | 1.5 |
| **9** | 19-25 Mar | 35.8 | 15.7 | 0.0 | 32.6 | 15.3 | 4.1 |
| **10** | 26 Mar-1 Apr | 36.3 | 17.3 | 0.0 | 33.7 | 16.6 | 3.8 |
| **11** | 2-8 Apr | 34.7 | 19.4 | 6.2 | 34.8 | 17.5 | 1.7 |
| **12** | 9-15 Apr | 35.1 | 20.1 | 1.4 | 36.8 | 18.7 | 2 |
| **13** | 16-22 Apr | 39.7 | 20.6 | 0.0 | 37.3 | 19.6 | 2.5 |
| **14** | 23-29 Apr | 32.7 | 18.9 | 0.0 | 38.3 | 21.1 | 4.1 |
| **Mean/**  **Total** |  | **31.7** | **14.9** | **15** | **30.4** | **14.0** | **56.8** |

2021

Kharif

| **Wk No.** | **Date** | **Tmax.**  **2021** | **Tmax.**  **Normal** | **Tmin.**  **2021** | **Tmax.**  **Normal** | **Rainfall**  **2021** | **Rainfall-Normal** |
| --- | --- | --- | --- | --- | --- | --- | --- |
| **21** | **May 21-27** | 38.9 | 42.5 | 27.2 | 27.4 | 0.0 | 5.3 |
| **22** | **May 28-03 Jun** | 40.2 | 42.3 | 27.8 | 27.6 | 0.0 | 6.6 |
| **23** | **Jun 04-10** | 39.3 | 40.6 | 26.1 | 27.5 | 107.8 | 17.5 |
| **24** | **11-17** | 30.8 | 38.0 | 25.1 | 26.2 | 41.4 | 47.5 |
| **25** | **18-24** | 32.3 | 34.8 | 24.8 | 25.2 | 54.4 | 59.3 |
| **26** | **25-01** | 33.1 | 32.8 | 25.5 | 24.6 | 42.6 | 71.4 |
| **27** | **Jul 02-08** | 33.6 | 32.3 | 25.1 | 24.4 | 135.0 | 64.9 |
| **28** | **09-15** | 32.1 | 31.2 | 26.0 | 24.1 | 16.2 | 84.8 |
| **29** | **16-22** | 34.2 | 30.5 | 26.5 | 24.1 | 56.8 | 82.8 |
| **30** | **23-29** | 30.4 | 30.4 | 24.8 | 24.1 | 88.2 | 71.7 |
| **31** | **30-05 Aug** | 28.8 | 30.3 | 24.8 | 24.2 | 15.8 | 86.2 |
| **32** | **Aug 06-12** | 33.0 | 29.8 | 25.6 | 24.1 | 63.6 | 77.8 |
| **33** | **13-19** | 32.8 | 29.9 | 26.0 | 24.1 | 25.8 | 89.2 |
| **34** | **20-26** | 32.1 | 30.1 | 25.6 | 24.1 | 22.8 | 53.6 |
| **35** | **27-02 Sep** | 32.6 | 30.1 | 25.5 | 24.1 | 9.8 | 69.5 |
| **36** | **Sep 03-09** | 32.6 | 30.4 | 24.9 | 24 | 86.0 | 57.2 |
| **37** | **10-16** | 31.1 | 30.9 | 24.5 | 24 | 238.4 | 55.5 |
| **38** | **17-23** | 30.2 | 31.4 | 24.3 | 24.1 | 16.2 | 38.6 |
| **39** | **24-30** | 31.8 | 31.7 | 24.7 | 23.8 | 26.8 | 24.2 |
| **40** | **Oct 01-07** | 32.7 | 31.5 | 25.1 | 23.1 | 0.0 | 16.4 |
| **41** | **08-14** | 32.6 | 31.7 | 23.0 | 22.2 | 0.4 | 10.8 |
| **42** | **15-21** | 32.4 | 31.2 | 24.3 | 20.6 | 15.2 | 9.0 |
| **43** | **22-28** | 31.4 | 30.5 | 18.1 | 18.8 | 0.0 | 10.4 |
| **44** | **29-04 Nov** | 30.4 | 29.8 | 19.1 | 17.7 | 0.0 | 4.0 |
| **45** | **Nov 05-11** | 30.1 | 29.8 | 14.8 | 16.3 | 0.0 | 3.9 |
| **46** | **12-18** | 29.1 | 29.6 | 21.3 | 15.2 | 0.6 | 1.9 |
| **47** | **19-25** | 31.0 | 29.1 | 20.4 | 14 | 48.0 | 3.0 |
| **48** | **26-02 Dec** | 29.1 | 28.5 | 13.6 | 12.8 | 0.0 | 2.4 |
|  | **Mean / Total** | **32.5** | **32.2** | **23.7** | **22.6** | **1111.8** | **1125.4** |

Rabi

| **Week No.** | **Dates** | **Max. Temp. (2021-22)** | **Min. Temp. (2021-22)** | **Rainfall**  **(2021-22)** | **Max. Temp. (Normal)** | **Min. Temp. (Normal)** | **Rainfall (Normal)** |
| --- | --- | --- | --- | --- | --- | --- | --- |
|  |  |  |  |  |  |  |  |
| **46** | 3-9 Dec | 29.1 | 21.3 | 0.6 | 29.5 | 15.1 | 2.6 |
| **47** | 10-16 Dec | 31.0 | 20.4 | 48 | 29.0 | 13.9 | 2.6 |
| **48** | 17-23 Dec | 29.1 | 13.6 | 0.0 | 28.3 | 12.6 | 2.9 |
| **49** | 24-31 Dec | 28.7 | 17.3 | 0.0 | 27.8 | 11.5 | 0.5 |
| **50** | 1-7 Jan | 27.8 | 13.4 | 0.0 | 27.5 | 11.2 | 1.5 |
| **51** | 8-14 Jan | 25.7 | 8.4 | 0.0 | 27.3 | 10.6 | 0.8 |
| **52** | 15-21 Jan | 26.4 | 13.8 | 82.4 | 27.1 | 10.8 | 1.5 |
| **1** | 22-28 Jan | 18.3 | 15.0 | 0.0 | 26.6 | 10.1 | 2.2 |
| **2** | 29 Jan-4 Feb | 24.3 | 16.6 | 15.0 | 27.2 | 11.3 | 3.8 |
| **3** | 5-11 Feb | 25.3 | 12.2 | 0.0 | 27.7 | 11.5 | 2 |
| **4** | 12-18 Feb | 26.0 | 12.1 | 3.0 | 28.1 | 11.7 | 3.8 |
| **5** | 19-25 Feb | 27.9 | 10.7 | 0.0 | 28.8 | 12.7 | 4.3 |
| **6** | 26 Feb-4 Mar | 28.5 | 11.4 | 7.0 | 29.1 | 12.9 | 4.4 |
| **7** | 5 -11 Mar | 27.6 | 12.3 | 0.0 | 30.3 | 14.1 | 4.2 |
| **8** | 12-18 Mar | 31.9 | 14.2 | 0.0 | 31.5 | 14.5 | 1.5 |
| **9** | 19-25 Mar | 33.1 | 17.9 | 0.0 | 32.6 | 15.3 | 4.1 |
| **10** | 26 Mar-1 Apr | 33.8 | 16.1 | 0.0 | 33.7 | 16.6 | 3.8 |
| **11** | 2-8 Apr | 36.2 | 17.9 | 0.0 | 34.8 | 17.5 | 1.7 |
| **12** | 9-15 Apr | 38.5 | 21.5 | 0.0 | 36.8 | 18.7 | 2 |
| **13** | 16-22 Apr | 39.8 | 19.7 | 0.0 | 37.3 | 19.6 | 2.5 |
| **14** | 23-29 Apr | 40.7 | 24 | 0.0 | 38.3 | 21.1 | 4.1 |
| **Mean/**  **Total** |  | **30.0** | **15.7** | **156.0** | 30.4 | 14.0 | 56.8 |

2022

Kharif

| **Wk No.** | **Date** | **Tmax.**  **2022** | **Tmax.**  **Normal** | **Tmin.**  **2022** | **Tmin.**  **Normal** | **Rainfall**  **2022** | **Rainfall-Normal** |
| --- | --- | --- | --- | --- | --- | --- | --- |
| **21** | **May 21-27** | 41.0 | 42.5 | 25.4 | 27.4 | 18.0 | 5.3 |
| **22** | **May 28-03 Jun** | 42.4 | 42.3 | 26.4 | 27.6 | 5.4 | 6.6 |
| **23** | **Jun 04-10** | 45.1 | 40.6 | 29.3 | 27.5 | 0.0 | 17.5 |
| **24** | **11-17** | 40.4 | 38.0 | 27.6 | 26.2 | 2.8 | 47.5 |
| **25** | **18-24** | 34.1 | 34.8 | 25.0 | 25.2 | 22.2 | 59.3 |
| **26** | **25-01** | 35.0 | 32.8 | 25.8 | 24.6 | 39.4 | 71.4 |
| **27** | **Jul 02-08** | 32.6 | 32.3 | 25.4 | 24.4 | 79.2 | 64.9 |
| **28** | **09-15** | 31.6 | 31.2 | 25.0 | 24.1 | 109.6 | 84.8 |
| **29** | **16-22** | 29.7 | 30.5 | 25.1 | 24.1 | 97.6 | 82.8 |
| **30** | **23-29** | 31.6 | 30.4 | 25.6 | 24.1 | 29.2 | 71.7 |
| **31** | **30-05 Aug** | 33.8 | 30.3 | 25.7 | 24.2 | 40.6 | 86.2 |
| **32** | **Aug 06-12** | 30.5 | 29.8 | 24.5 | 24.1 | 212.4 | 77.8 |
| **33** | **13-19** | 30.0 | 29.9 | 25.0 | 24.1 | 104.6 | 89.2 |
| **34** | **20-26** | 30.9 | 30.1 | 24.6 | 24.1 | 43.0 | 53.6 |
| **35** | **27-02 Sep** | 33.3 | 30.1 | 25.5 | 24.1 | 67.0 | 69.5 |
| **36** | **Sep 03-09** | 32.4 | 30.4 | 25.5 | 24.0 | 32.4 | 57.2 |
| **37** | **10-16** | 30.6 | 30.9 | 24.8 | 24.0 | 39.4 | 55.5 |
| **38** | **17-23** | 30.8 | 31.4 | 24.2 | 24.1 | 73.2 | 38.6 |
| **39** | **24-30** | 31.5 | 31.7 | 24.0 | 23.8 | 26.0 | 24.2 |
| **40** | **Oct 01-07** | 31.6 | 31.5 | 24.5 | 23.1 | 11.8 | 16.4 |
| **41** | **08-14** | 31.3 | 31.7 | 23.9 | 22.2 | 40.0 | 10.8 |
| **42** | **15-21** | 31.6 | 31.2 | 22.8 | 20.6 | 7.4 | 9.0 |
| **43** | **22-28** | 30.8 | 30.5 | 17.5 | 18.8 | 0.0 | 10.4 |
| **44** | **29-04 Nov** | 30.2 | 29.8 | 16.2 | 17.7 | 0.0 | 4.0 |
| **45** | **Nov 05-11** | 31.5 | 29.8 | 16.7 | 16.3 | 0.0 | 3.9 |
| **46** | **12-18** | 30.0 | 29.6 | 13.8 | 15.2 | 0.0 | 1.9 |
| **47** | **19-25** | 28.8 | 29.1 | 11.8 | 14.0 | 0.0 | 3.0 |
| **48** | **26-02 Dec** | 29.5 | 28.5 | 11.2 | 12.8 | 0.0 | 2.4 |
|  | **Mean / Total** | **33.0** | **32.2** | **23.0** | **22.6** | **1101.2** | **1125.4** |

Rabi

| **Week No.** | **Dates** | **Max. Temp. (2022-23)** | **Min. Temp. (2022-23)** | **Rainfall**  **(2022-23)** | **Max. Temp. (Normal)** | **Min. Temp. (Normal)** | **Rainfall (Normal)** |
| --- | --- | --- | --- | --- | --- | --- | --- |
|  |  |  |  |  |  |  |  |
| **46** | Nov 12-18 | 30.0 | 13.8 | 0.0 | 29.5 | 15.1 | 2.6 |
| **47** | 19-25 | 28.8 | 11.8 | 0.0 | 29.0 | 13.9 | 2.6 |
| **48** | 26-02 | 29.5 | 11.2 | 0.0 | 28.3 | 12.6 | 2.9 |
| **49** | Dec 03-09 | 28.8 | 12.0 | 0.0 | 27.8 | 11.5 | 0.5 |
| **50** | 10-16 | 29.7 | 15.2 | 0.0 | 27.5 | 11.2 | 1.5 |
| **51** | 17-23 | 29.4 | 11.1 | 0.0 | 27.3 | 10.6 | 0.8 |
| **52** | 24-31 | 31.0 | 12.9 | 0.0 | 27.1 | 10.8 | 1.5 |
| **1** | Jan 01-07 | 26.4 | 13.4 | 1.8 | 26.6 | 10.1 | 2.2 |
| **2** | 08-14 | 29.3 | 9.2 | 0.0 | 27.2 | 11.3 | 3.8 |
| **3** | 15-21 | 29.6 | 14.0 | 0.0 | 27.7 | 11.5 | 2 |
| **4** | 22-28 | 32.1 | 14.6 | 0.0 | 28.1 | 11.7 | 3.8 |
| **5** | 29-04 | 30.7 | 13.7 | 0.0 | 28.8 | 12.7 | 4.3 |
| **6** | Feb 05-11 | 32.2 | 11.5 | 0.0 | 29.1 | 12.9 | 4.4 |
| **7** | 12-18 | 32.7 | 12.2 | 0.0 | 30.3 | 14.1 | 4.2 |
| **8** | 19-25 | 34.9 | 14.2 | 0.0 | 31.5 | 14.5 | 1.5 |
| **9** | 26-04 | 35.7 | 16.4 | 0.0 | 32.6 | 15.3 | 4.1 |
| **10** | Mar 05-11 | 35.4 | 17.4 | 1.2 | 33.7 | 16.6 | 3.8 |
| **11** | 12-18 | 35.2 | 19.9 | 0.0 | 34.8 | 17.5 | 1.7 |
| **12** | 19-25 | 31.4 | 18.6 | 14.8 | 36.8 | 18.7 | 2 |
| **13** | 26-01 | 36.8 | 20.1 | 6.8 | 37.3 | 19.6 | 2.5 |
| **14** | Apr 02-08 | 36.3 | 21.3 | 0.0 | 38.3 | 21.1 | 4.1 |
| **Mean/**  **Total** |  | **31.7** | **14.5** | **24.6** | **30.4** | **14.0** | **56.8** |
